# Shear-induced Notch-Cx37-p27 axis arrests endothelial cell cycle to enable arterial specification

**DOI:** 10.1038/s41467-017-01742-7

**Published:** 2017-12-15

**Authors:** Jennifer S. Fang, Brian G. Coon, Noelle Gillis, Zehua Chen, Jingyao Qiu, Thomas W. Chittenden, Janis M. Burt, Martin A. Schwartz, Karen K. Hirschi

**Affiliations:** 1 Department of Medicine, Yale University School of Medicine, 333 Cedar Street, New Haven, CT 06520 USA; 2 Yale Cardiovascular Research Center, Yale University School of Medicine, 333 Cedar Street, New Haven, CT 06520 USA; 3 Vascular Biology and Therapeutics Program, Yale University School of Medicine, 333 Cedar Street, New Haven, CT 06520 USA; 4 Yale Stem Cell Center, Yale University School of Medicine, 333 Cedar Street, New Haven, CT 06520 USA; 5 Computational Statistics and Bioinformatics Group, Advanced Artificial Intelligence Research Laboratory, WuXi NextCODE 55 Cambridge Parkway, 8th Floor, Cambridge, MA 02142 USA; 6 Department of Genetics, Yale University School of Medicine, 333 Cedar Street, New Haven, CT 06520 USA; 7 Division of Genetics and Genomics, Boston Children’s Hospital, Harvard Medical School, A-111, 25 Shattuck Street, Boston, MA 02115 USA; 8 Department of Biological Engineering, Massachusetts Institute of Technology, 21 Ames Street #56-651, Cambridge, MA 02142 USA; 9 Department of Physiology, College of Medicine, The University of Arizona, 1501 N. Campbell Road, Tucson, AZ 85724 USA; 10 Department of Cell Biology, Yale University School of Medicine, 333 Cedar Street, New Haven, CT 06520 USA; 11 Department of Biomedical Engineering, Yale University School of Medicine, 333 Cedar Street, New Haven, CT 06520 USA

## Abstract

Establishment of a functional vascular network is rate-limiting in embryonic development, tissue repair and engineering. During blood vessel formation, newly generated endothelial cells rapidly expand into primitive plexi that undergo vascular remodeling into circulatory networks, requiring coordinated growth inhibition and arterial-venous specification. Whether the mechanisms controlling endothelial cell cycle arrest and acquisition of specialized phenotypes are interdependent is unknown. Here we demonstrate that fluid shear stress, at arterial flow magnitudes, maximally activates NOTCH signaling, which upregulates GJA4 (commonly, Cx37) and downstream cell cycle inhibitor CDKN1B (p27). Blockade of any of these steps causes hyperproliferation and loss of arterial specification. Re-expression of GJA4 or CDKN1B, or chemical cell cycle inhibition, restores endothelial growth control and arterial gene expression. Thus, we elucidate a mechanochemical pathway in which arterial shear activates a NOTCH-GJA4-CDKN1B axis that promotes endothelial cell cycle arrest to enable arterial gene expression. These insights will guide vascular regeneration and engineering.

## Abstract (web summary)

New vessel formation relies on a tightly controlled switch in endothelial biology from proliferating to  specializing phenotypes. Here, Fang et al. elucidate the molecular mechanisms of this switch and show that the arterial shear activates a Notch-Cx37-p27 axis promoting endothelial cell cycle arrest and enabling arterial gene expression.

## Introduction

Establishment of a well-organized and perfused circulatory system is essential to oxygenate tissues and remove metabolic waste. When new blood vessels form, during development or in response to tissue injury, newly generated endothelial cells rapidly proliferate and coalesce into disorganized capillary plexi. Coincident with the onset of blood flow through vessel lumens, endothelial cell proliferation is reduced and primitive vessels remodel into arterial-venous networks that acquire mural cell coverage (reviewed in Ribatti et al.^1^). Although we have made progress in identifying factors that stimulate endothelial cell proliferation and sprouting (reviewed in Marcelo 2013a^2^), limited understanding of the regulation of endothelial cell growth suppression and phenotypic specialization during vascular remodeling remains a significant roadblock for clinical therapies, tissue engineering and regenerative medicine.

Fluid shear stress (FSS) likely guides vascular remodeling to maximize efficient tissue perfusion (reviewed in Baeyens and Schwartz, 2015^3^), but underlying mechanisms are poorly understood. Interestingly, both flow-induced mechanotransduction^4–10^ and NOTCH signaling^11–15^ are implicated in endothelial growth control and arterial development; however, whether these pathways coordinately regulate these processes, and whether endothelial cell growth arrest is required for arterial-venous specification, require further study.

We recently found that endothelial cells require NOTCH-induced cell cycle arrest via regulation of CDKN1B (commonly, p27) for acquisition of a hemogenic phenotype that enables blood-forming potential^16^. Since NOTCH is also implicated in arterial^11^, as well as lymphatic^17^, endothelial cell development, we considered whether NOTCH might play a common role in these processes. That is, perhaps NOTCH-induced cell cycle arrest is required for endothelial cells to acquire all of these specialized phenotypes and functions. Indeed, cell cycle state of undifferentiated embryonic stem cells strongly influences cell fate decisions^18^, but it is unclear whether a similar mechanism applies to endothelial cell specification. We, therefore, investigated whether NOTCH signaling mediates flow-induced endothelial cell growth control, and whether endothelial cell cycle state determines their propensity to acquire an arterial identity.

Examining both post-natal retina neovascularization and cultured endothelial cells, we define a novel signaling pathway whereby FSS, at arterial magnitudes, maximally activates NOTCH signaling, which upregulates GJA4, more commonly known as Connexin37 (Cx37), and downstream CDKN1B to promote endothelial G1 arrest and to enable expression of arterial genes. This link between endothelial cell cycle and cell fate was not previously known, and is critically important for controlling blood vessel development and remodeling. Insights gained from these studies will facilitate efforts to optimize vascular regeneration of injured and diseased tissues in vivo and blood vessel engineering ex vivo.

## Results

### Flow-dependent endothelial quiescence is mediated by NOTCH

**Fig. 1:**
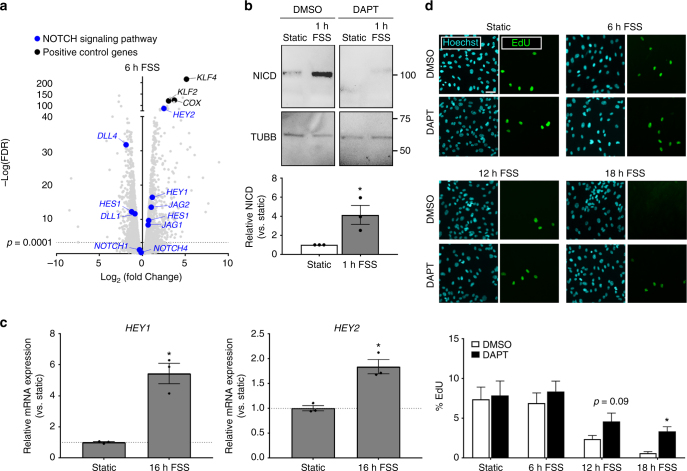
NOTCH signaling regulates shear-induced endothelial cell quiescence. **a** Expression of several NOTCH signaling pathway effectors were significantly altered in whole-transcriptome analysis of HUVEC exposed to 6 h FSS (vs. 6 h Static), as were previously characterized flow-responsive genes *KLF2*, *KLF4*, and *COX*. **b** Cleavage of NOTCH receptor intracellular domain (NICD) was significantly increased with 1 h of 12 dynes/cm^2^ FSS (mean densitometry ± SEM; *n* = 3 for all groups; Students’ *t*-test: *p* = 0.03), and blocked by 10 µM DAPT. Uncropped blots presented in Supplementary Fig. 1. **c** *HEY1* and *HEY2* transcript levels were elevated with 16 h FSS (mean relative mRNA expression ± SEM vs. Static; *n* = 3 for all groups; Students’ *t*-test: *p* = 0.003 (*HEY1*), *p* = 0.005 (*HEY2*)). **d** EdU incorporation, a measure of DNA synthesis and indicator of proliferation, was reduced over 18 h exposure to FSS in control cells, and this response was abrogated by inhibition of NOTCH signaling (via 10 µM DAPT) (Colors: Hoechst (cyan), EdU (green); scale bar = 100 µm; mean % EdU-positive nuclei ± SEM for 8 ROI; representative of *n* = 3 experiments; Students’ *t*-test: *p* = 0.09 (12 h), *p* = 0.0006 (18 h))

Preliminary experiments confirmed that physiological FSS (12 dynes/cm^2^) suppressed the incorporation of EdU, a measure of DNA synthesis and indicator of proliferation, in human umbilical vein endothelial cells (HUVEC) at 12–24 h. To identify mediators of flow-dependent endothelial cell quiescence, we performed whole-transcriptome sequencing (RNA-seq) on HUVEC under static or FSS conditions for 6 h, a time likely to reveal cell signaling pathways that mediate cell cycle arrest following onset of shear. FSS altered the expression of 6,512 genes. Gene ontology (GO) and nested gene ontology (nGO) analyses designed to control for gene length bias were used to assess functional enrichment of altered genes, and a subset of GO-nGO pairs were selected for overlapping relevance to cell proliferation, cell signaling and development (Supplementary Data 1). NOTCH signaling was the top candidate pathway within this subset (Supplementary Table 1). Several NOTCH-associated genes, including ligands *DLL1*, *DLL4*, *JAG1*, and *JAG2*, and canonical NOTCH transcriptional targets *HES1*, *HES2*, *HEY1*, and *HEY2*, were altered by shear (Fig. 1a). Interestingly, mRNA levels for endothelial-expressed receptors *NOTCH1* and *NOTCH4* were not affected by FSS. Activation of shear-dependent signaling was confirmed by strong upregulation of *KLF2*, *KLF4*, and *COX* genes.

To verify that flow induces NOTCH signaling, HUVEC were exposed to shear. Cleavage of the NOTCH intracellular domain (NICD), which mediates gene transcription upon receptor activation, was increased with 1 h FSS and blocked by pre-treatment with 10 µM DAPT, a pharmacological inhibitor of NOTCH cleavage and signaling (Fig. 1b and Supplementary Fig. 1). In addition, NOTCH target genes *HEY1* and *HEY2* were significantly upregulated by 16 h of FSS (Fig. 1c). Inhibiting NICD cleavage with 10 µM DAPT also significantly alleviated FSS-mediated suppression of endothelial cell EdU incorporation (Fig. 1d). Altogether, these data show that NOTCH signaling mediates shear-induced endothelial cell growth suppression.

### GJA4 mediates endothelial quiescence downstream of NOTCH

**Fig. 2:**
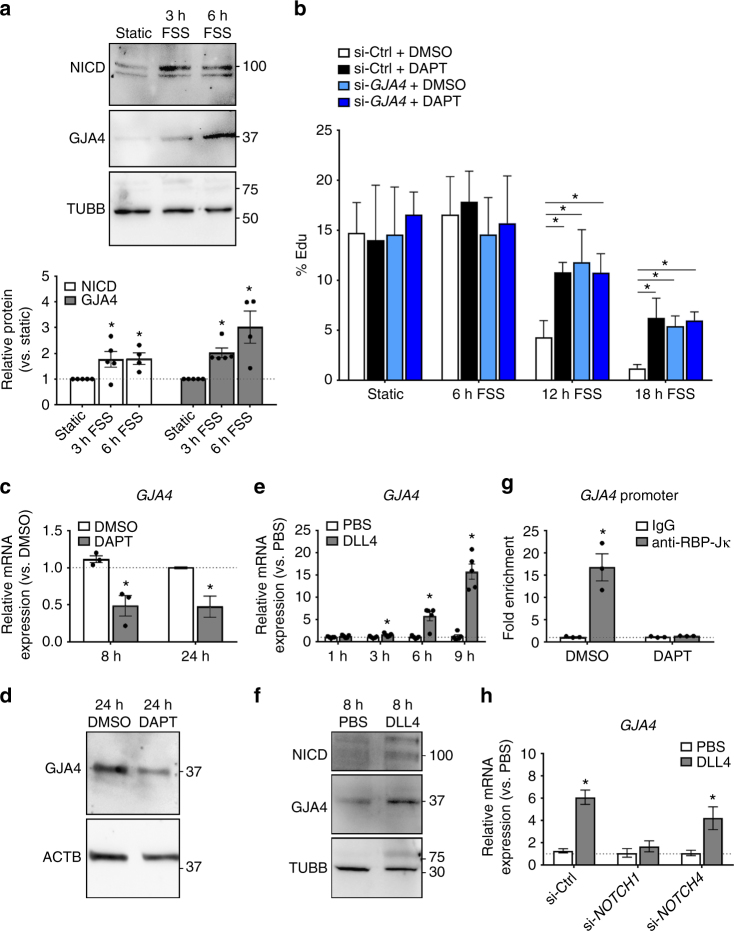
Shear-sensitive NOTCH upregulates GJA4 expression. **a** Exposure of HUVEC to 6 h FSS significantly upregulated GJA4 protein, in parallel with sustained increase in NICD (mean densitometry ± SEM; *n* = 5 (Static, 3 h FSS), *n* = 4 (6 h FSS); one-way ANOVA: p = 0.04 (NICD), *p* = 0.004 (GJA4), asterisks indicate *p* < 0.05 in post hoc *t*-test vs. Static). Uncropped blots presented in Supplementary Fig. 3. **b** si-*GJA4* and DAPT (alone or in combination) abrogated flow-induced suppression of EdU incorporation at 12–18 h shear (mean % EdU-positive nuclei ± SEM for 8 ROI; representative of *n* = 3 experiments; one-way ANOVA: *p* < 0.0001 (12 h FSS, 18 h FSS), asterisks indicate *p *< 0.05 in post hoc *t*-test). **c** *GJA4* levels were reduced by treatment of confluent HUVEC with 8 h or 24 h DAPT (mean relative mRNA expression ± SEM vs. DMSO; *n* = 3 (8 h), *n* = 5 (24 h); individual values plotted where *n* < 5; Students’ *t*-test: *p* = 0.01 (8 h), *p* = 0.006 (24 h)). **d** 24 h DAPT also reduced GJA4 protein levels in confluent HUVEC. Uncropped blots presented in Supplementary Fig. 3. **e** Seeding of sub-confluent HUVEC onto recombinant DLL4 increased *GJA4* mRNA levels (mean relative mRNA expression ± SEM vs. PBS; *n* = 5 for all groups; Students’ *t*-test: *p* = 0.03 (3 h), *p* = 0.002 (6 h), *p* < 0.0001 (9 h)). **f** GJA4 protein and NICD were increased following seeding of sub-confluent HUVEC onto Dll4 for 8 h. Uncropped blots presented in Supplementary Fig. 3. **g** Significant *GJA4* promoter fold enrichment was detected in samples assayed by ChIP using anti-RBP-Jκ, compared to IgG controls. This effect was abolished with DAPT treatment (mean fold enrichment ± SEM vs IgG; *n* = 3 for all groups; Students *t*-test: *p* = 0.007 (anti-RBP-Jκ)). **h** si-*NOTCH1*, but not si-*NOTCH4*, abolished DLL4-induced upregulation of *GJA4* mRNA at 6 h following cell seeding (mean relative mRNA expression ± SEM vs. PBS; *n *= 7 (si-Ctrl, si-*NOTCH1*), *n* = 8 (si-Ctrl, si-*NOTCH4*); Students’ *t*-test: *p* < 0.0001 (si-Ctrl), *p* = 0.02 (si-*NOTCH4*))

To identify genes regulated by NOTCH under flow, RNA-seq was performed on HUVEC exposed to 6 h of FSS with or without 10 µM DAPT. Of the top five genes, only *GJA4* (which encodes GJA4, more commonly known as Connexin37, or Cx37) and *TMEM100* (which encodes Transmembrane Protein 100) were both upregulated by shear and suppressed in the presence of DAPT (Supplementary Table 2 and Supplementary Fig. 2). Of these, GJA4 was selected for further study because of its endothelial-specific expression during development^19^, and in vivo evidence of its involvement in blood^20,21^ and lymphatic^17,22–24^ vascular morphogenesis. We found that GJA4 protein was elevated after 6 h FSS in association with sustained upregulation of NICD (Fig. 2a and Supplementary Fig. 3). GJA4 knockdown (si-*GJA4*, validated in Supplementary Fig. 4) impaired shear-induced suppression of cell proliferation, as did  DAPT (si-Ctrl+DAPT), while si-*GJA4* combined with DAPT had no additional effects (Fig. 2b). Altogether, these data suggest that GJA4 is the predominant mediator of NOTCH- and flow-induced endothelial cell growth arrest.

### NOTCH signaling regulates GJA4 expression

To determine whether NOTCH regulates GJA4, we used confluent endothelial cell monolayers as a convenient system, in which there is basal activation of NOTCH through ligand–receptor interactions. As expected, endothelial cells exhibited density-dependent NOTCH activation, which was suppressed by DAPT inhibition of NICD cleavage (Supplementary Fig. 5), resulting in decreased endogenous *HES1* mRNA levels (Supplementary Fig. 6A). DAPT treatment of confluent endothelial cells also reduced mRNA and protein levels for GJA4 (Fig. 2c, d and Supplementary Fig. 3). Basal NOTCH signaling was undetectable in sub-confluent HUVEC (Supplementary Fig. 5), but induced by seeding sub-confluent cells onto immobilized recombinant DLL4 ligand to constitutively activate NOTCH leading to upregulation of *HES1* mRNA (Supplementary Fig. 4B). Seeding of sub-confluent HUVEC onto recombinant DLL4 also upregulated *GJA4* mRNA and protein, along with increased NICD (Fig. 2e, f).

NOTCH stimulates transcription of target genes through binding of cleaved NICD to a nuclear gene regulatory complex that includes the DNA-binding protein RBP-Jκ. In silico analysis revealed several high-probability RBP-Jκ binding motifs in the regulatory regions of the mouse and human *GJA4* genes including several motifs adjacent to the transcriptional start site (Supplementary Table 3). To determine whether *GJA4* is a direct target of the NICD-RBP-Jκ complex, we performed chromatin-immunoprecipitation (ChIP) on confluent HUVEC monolayers (in which NOTCH is basally active, Supplementary Fig. 5). Immunoprecipitates of RBP-Jκ from these cells revealed enrichment of the *GJA4* promoter region relative to the IgG control, which was blocked by DAPT (Fig. 2g). Thus, GJA4 appears to be a transcriptional target of NICD/RBP-Jκ.

Endothelial cells express two NOTCH receptors, NOTCH1 and NOTCH4, both capable of inducing canonical NOTCH signaling and downstream transcriptional activation. Expression of each NOTCH receptor was selectively reduced by siRNA against *NOTCH1* or *NOTCH4*, respectively (Supplementary Fig. 6C). Knockdown of either NOTCH1 or NOTCH4 abolished DLL4-induced upregulation of *HES1* (Supplementary Fig. 6D); however, only si-*NOTCH1*, and not si-*N* *O* *T* *C* *H* *4*, eliminated DLL4-induced upregulation of *GJA4* mRNA (Fig. 2h), suggesting that GJA4 expression is specifically regulated by NOTCH1.

### GJA4 is expressed in remodeling blood vessel endothelium

To examine the role of GJA4 in vascular remodeling in vivo, we used the well-studied neonatal murine retinal vascularization model^12,25–27^. Beginning at post-natal day 1 (P1), rapid, highly stereotyped vascularization initiated from a central vascular plexus can be visualized over subsequent days by labeling endothelial cells using either antibodies targeting PECAM1 (or, CD31) or with fluorescently conjugated isolectin B4 (IB4). (Supplementary Fig. 7A). Over subsequent days, endothelial cells at the retinal vascular edge proliferate and migrate radially outward, while nascent blood vessels interior from the vascular front remodel, mature and recruit smooth muscle actin (SMA)-positive mural cells to form a functional circulatory network. At P6, the developing retinal vasculature includes an outer region of active vessel remodeling (R) and a more central region with mature (M), specified arteries and veins. For our studies, we designated the R–M boundary (dashed yellow line) as the distance of SMA investment of arteries, (Supplementary Fig. 7B), which was 519 ± 64μm (n = 6, multiple litters) from the vascular front at P6. Intracardiac injection of rhodamine dextran revealed that vessels of the remodeling plexus were lumenized and were accessible to systemic blood flow (Supplementary Fig. 7C). This finding was consistent with in silico models of retinal blood flow, which show that the remodeling plexi experience near-arterial levels of shear stress^28^. In addition, activated ERK5 (pERK5), which is acutely induced by flow in endothelial cells in culture^29^, was abundant in remodeling retinal vessels (Supplementary Fig. 7D).

**Fig. 3:**
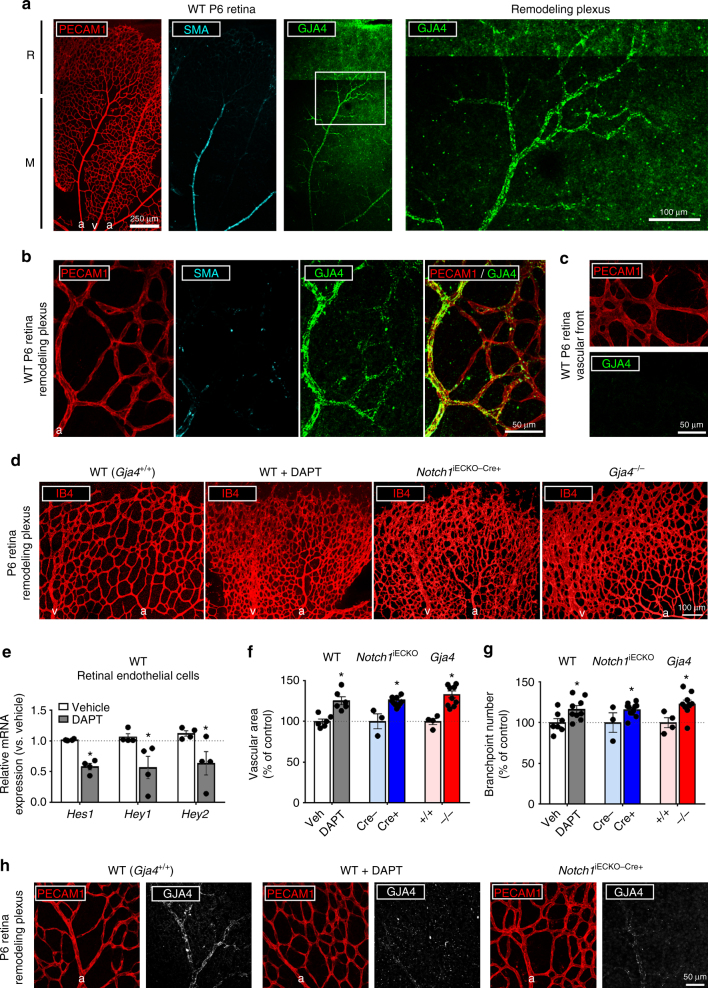
GJA4 regulates vascular remodeling. **a** GJA4 expression was predominantly detected in the remodeling plexus at the R-M boundary, in addition to expression in SMA-invested arteries. (Colors: PECAM1 (red), SMA (cyan), GJA4 (green); scale bar: 250 µm (lower magnification), 100 µm (higher magnification); symbols: a = artery, v = vein). **b** At higher magnification, GJA4 expression was observed in vessels beyond the R-M boundary, including in large vessels not invested with SMA, as well as in adjacent smaller vessels. (Colors: PECAM1 (red), SMA (cyan), GJA4 (green); Scale bar = 50 µm; Symbols: a = artery). **c** GJA4 was absent from tip cells at the vascular edge. (Colors: PECAM1 (red), GJA4 (green); Scale bar = 50 µm). **d** In P6 retinas lacking GJA4 (*Gja4* ^−/−^) or NOTCH (WT + DAPT, or *Notch1* ^iECKO−Cre+^), the developing vasculature was hyperdense in comparison to wild-type (*Gja4* ^+/+^) tissues. (Colors: IB4 (red); scale bar = 100 µm; symbols: a = artery, v = vein). **e** Expression of NOTCH target genes *HES1*, *HEY1*, and *HEY2* were significantly reduced in FACS-sorted endothelial cells (PECAM1+/PTPRC−, commonly CD31+/CD45–) with DAPT treatment (mean relative mRNA expression ± SEM vs. vehicle-treated controls; *n* = 4 for all groups; paired Students’ *t*-test: *p* = 0.0002 (*Hes1*), *p* = 0.04 (*Hey1*), *p* = 0.047 (*Hey2*)). **f** Vessel (PECAM1+ or IB4+) area was significantly increased in NOTCH-inhibited (WT + DAPT or *Notch1* ^iECKO−Cre+^) and GJA4-deleted (*Gja4* ^−/−^) mice compared to associated controls (mean % vessel area per retina ± SEM vs. control; *n* = 6 (WT + Veh, WT + DAPT), *n* = 3 (*Notch1* ^iECKO−Cre−^), *n* = 11 (*Notch1* ^iECKO−Cre+^), *n* = 4 (*Gja4* ^+/+^), *n* = 10 (*Gja4* ^−/−^); Students’ *t*-test: *p* = 0.001 (WT + Veh vs. WT + DAPT), *p* = 0.004 (*Notch1* ^iECKO−Cre−^ vs. *Notch1* ^iECKO−Cre+^), *p* = 0.0006 (*Gja4* ^*+/+*^ *vs. Gja4* ^−/−^)). **g** Branchpoint number was also significantly increased in WT + DAPT, *Notch1* ^iECKO−Cre+^, and *Gja4* ^−/−^ animals (mean % branchpoint number per retina ± SEM vs. control; *n* = 7 (WT + Veh), *n* = 9 (WT + DAPT), *n* = 3 (*Notch1* ^iECKO−Cre−^), *n* = 11 (*Notch1* ^iECKO−Cre+^), *n* = 4 (*Gja4* ^+/+^), *n* = 9 (*Gja4* ^−/−^) Students’ *t*-test: *p* = 0.03 (WT + Veh vs. WT + DAPT), *p* = 0.049 (*Notch1* ^iECKO−Cre−^ vs. *Notch1* ^iECKO−Cre+^), *p* = 0.002 (*Gja4* ^*+/+*^ *vs. Gja4* ^−/−^)). **h** GJA4 expression was significantly reduced in remodeling vessels of DAPT-treated WT animals, as well as in *Notch1* ^iECKO−Cre+^ animals. (Colors: PECAM1 (red), GJA4 (white); scale bar = 50 µm; symbols: a = artery)

In wild-type (WT) P6 mice, GJA4 was expressed in endothelial cells of the remodeling vessel plexus and in associated maturing arteries (Fig. 3a, b), but not at the vascular edge (Fig. 3c) where FSS is limited^28^ and where endothelial cells are highly proliferative^13^. In adult tissues, GJA4 is typically observed co-expressed with GJA5 (commonly, Connexin40 or Cx40) in large arteries;^30,31^ however, in the developing neonatal mouse retina, GJA5 was restricted to SMA-positive arteries (Supplementary Fig. 8), and not detected in remodeling vessels at the R–M boundary where GJA4 was highly expressed (Fig. 3b).

### Loss of GJA4 or NOTCH signaling causes remodeling defects

GJA4 is implicated in blood vessel formation, as mice deficient in GJA4 exhibit increased tissue vascularization[20], enhanced neovascularization of injured tissues^20,32^, and dysregulation of venous valve formation^21^. However, developmental blood vessel formation has not been well studied in these mice. We therefore examined vascularization of *Gja4* ^−/−^, *Gja4* ^+/−^, and *Gja4* ^+/+^ (WT) neonatal retinas. Vascular morphology was similar between *Gja4* ^+/−^ and age-matched *Gja4* ^+/+^ controls (Supplementary Fig. 9A–C). However, *Gja4* ^−/−^ retinal vascular plexi were hyperdense and poorly remodeled (Fig. 3d) compared to age-matched *Gja4* ^+/+^ controls, and there was a ~25% increase in vascular area and vessel branchpoint number within the remodeling zone of *Gja4* ^−/−^ mice (Fig. 3f–g). Hyperdensity was observed in nearly 80% of imaged remodeling plexi from *Gja4* ^−/−^ retinas, indicating high penetrance of this phenotype (Supplementary Fig. 9D). Consistent with the absence of detectible GJA4 in endothelial cells at the vascular edge, GJA4 deletion had no effect on tip cell number (Supplementary Fig. 10).

The *Gja4* ^−/−^ phenotype resembles the hyperdense vasculature observed with disrupted NOTCH signaling^12,25^, such as with DAPT treatment of WT mice or with tamoxifen-induced endothelial-specific deletion of NOTCH1 (*Notch1* ^iECKO^) (Fig. 3d). Pharmacological inhibition of endothelial NOTCH signaling via 24 h DAPT was confirmed by observation of a ~50% decrease in expression of canonical NOTCH target genes (*Hes1*, *Hey1* and *Hey2*) in retinal endothelial cells (Fig. 3e). Like *Gja4* ^−/−^ animals, DAPT-treated WT and *Notch1* ^iECKO^-Cre+ animals experienced a ~25% increase in vascular area and branchpoint number in the remodeling zone (Fig. 3f, g) compared to associated controls. However, unlike with GJA4 deletion, NOTCH inhibition (via DAPT) uniquely increased tip cell number (Supplementary Fig. 10), suggesting that GJA4 mediates distinct NOTCH effects in the remodeling vasculature. Consistent with this, GJA4 expression was greatly reduced in remodeling vessels of both DAPT-treated WT and *Notch1* ^iECKO^ retinas (Fig. 3h).

To determine whether *Gja4* ^−/−^ retinas exhibited vascular morphology defects at later stages of development, we assessed the retinal vasculature at P21, at which time the superficial vascular plexus established in P3-9 (Supplementary Fig. 7A) has undergone additional remodeling and regression to form a mature network. Meanwhile, smaller vessels have sprouted from the superficial vasculature beginning at P10, and have invaded downward to form a secondary deep vascular plexus below the superficial network. Consistent with our observations at P6, we observed a significant increase in vascular area in the deep vascular plexus of *Gja4* ^−/−^ mice compared to *Gja4* ^+/−^ littermates (Supplementary Fig. 11A). In addition, we observed a significant increase in the number of arterial branches in the superficial plexus of *Gja4* ^−/−^ compared to littermate controls (Supplementary Fig. 11B) even though overall vascular area was not measurably different at this stage (Supplementary Fig. 11A). These data suggest that while many of the vessels of the hyperdense *Gja4* ^−/−^ remodeling plexus (Fig. 3d) may eventually regress, *Gja4* ^−/−^ animals retain a vascular hyperbranching phenotype in the mature retinal circulation, which is consistent with our previous observations of excessive collateral connections in the pial circulation and increased hindlimb vascularization in response to ischemic damage^20,32^.

### NOTCH suppresses endothelial cell proliferation via GJA4

**Fig. 4:**
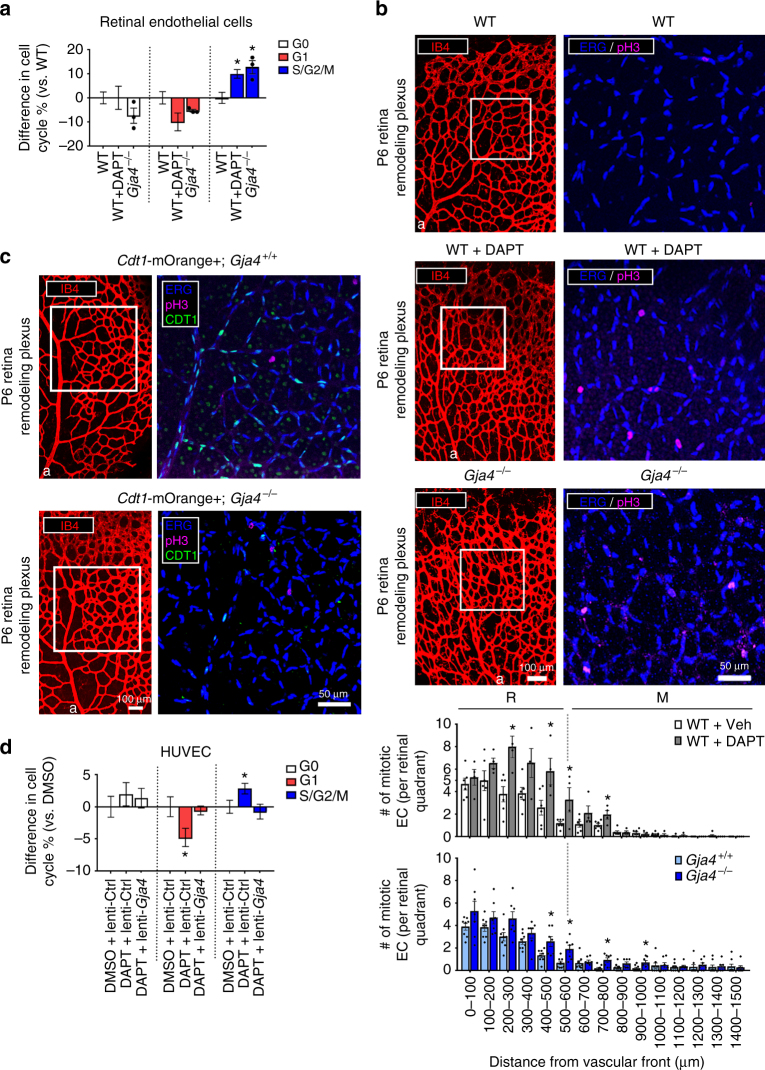
NOTCH regulates endothelial cell cycle progression via GJA4. **a** In endothelial (CD31+/CD45−) cells isolated from WT + DAPT and *Gja4* ^−/−^ P6 retinas, there was a decreased proportion in G0 or G1, and increased proportion in S/G2/M, compared to WT controls (mean difference in cell cycle phase % ± SEM vs. WT (*Gja4* ^+/+^); *n* = 6 (WT), *n* = 12 (WT + DAPT), *n* = 3 (*Gja4* ^−/−^); individual values plotted where *n* < 5; one-way ANOVA: *p* = 0.005 (S/G2/M), asterisks indicate *p* < 0.05 in post hoc *t*-tests). **b** pH3+ endothelial cells were more numerous in remodeling vessels of WT + DAPT and *Gja4* ^−/−^ P6 retinas, compared to controls, and mitotic endothelial cells were mostly found at the R-M boundary (Colors: IB4 (red), ERG (blue), pH3 (magenta); scale bar: 100 µm (low magnification), 50 µm (high magnification); symbols: a = artery, r = remodeling, m = mature; mean number of pH3+/ERG+ nuclei ± SEM vs control; *n* = 8 (*Gja4* ^+/+^, *Gja4* ^−/−^), *n* = 6 (WT), *n *= 4 (WT + DAPT); Students’ *t*-test: *p* = 0.006 (WT + Veh vs. WT + DAPT, 200–300 µm), *p* = 0.03 (WT + Veh vs. WT + DAPT, 400–500 µm), *p* = 0.04 (WT + Veh vs. WT + DAPT. 500–600 µm), *p* = 0.04 (WT + Veh vs. WT + DAPT, 700–800 µm), *p* = 0.04 (*Gja4* ^+/+^ vs. *Gja4* ^−/−^, 200–300 µm, *p* = 0.02 (*Gja4* ^+/+^ vs. *Gja4* ^−/−^, 400–500 µm), *p* = 0.005 (*Gja4* ^+/+^ vs. *Gja4* ^−/−^, 500–600 µm), *p* = 0.005 (*Gja4* ^+/+^ vs. *Gja4* ^−/−^, 700–800 µm), *p* = 0.03 (*Gja4* ^+/+^ vs. *Gja4* ^−/−^, 900–1000 µm)). **c** The nuclei of endothelial cells in G1 were marked by CDT1-mOrange in remodeling vessels of *Cdt1*-mOrange+;*Gja4* ^+/+^ reporter mice. CDT1-mOrange signal in endothelial cells of remodeling vessels in *Gja4* ^−/−^ reporter mice was greatly reduced, compared to WT controls. (Colors: IB4 (red), ERG (blue), pH3 (magenta), CDT1 (green); scale bar: 100 µm (low magnification), 50 µm (high magnification); symbols: a = artery). **d** In HUVEC, DAPT decreased the proportion of cells in G1 and increased the proportion in S/G2/M; constitutive GJA4 expression (via lenti-*Gja4*) abolished these cell cycle effects of DAPT (mean difference in cell cycle % ± SEM vs. DMSO; *n* = 9 (DMSO), *n* = 7 (DAPT), *n* = 8 (DAPT+lenti-*Gja4*); one-way ANOVA: *p* = 0.04 (G1), *p* = 0.03 (S/G2/M), asterisks indicate *p* < 0.05 in post hoc *t*-tests)

The hyperdense phenotype of the NOTCH-inhibited and *Gja4* ^−/−^ P6 retinal vasculature (Fig. 3) suggested altered endothelial cell proliferation. Using fluorescence-activated cell sorting (FACS), we isolated retinal endothelial cells (CD31+/CD45−) from WT (*Gja4* ^+/+^), *Gja4* ^−/−^ and WT+DAPT P6 mice, and examined their cell cycle distribution. WT P6 retinal endothelial cells had the following cell cycle distribution: 27.0 ± 2.6% in G0, 50.6 ± 2.8% in G1 and 22.4 ± 2.5 % in S/G2/M. Both WT+DAPT and *Gja4* ^−/−^ retinas appeared to have fewer endothelial cells in G0 and G1 phases, and in both groups a significantly increased proportion of actively cycling (S/G2/M) cells were observed (Fig. 4a). We further confirmed an increase in endothelial cell proliferation using the mitotic marker phospho-HIST3 (pH3), in conjunction with endothelial nuclear marker (ERG) (Fig. 4b, images). We found increased numbers of pH3+ endothelial cell nuclei (pH3+ /ERG+ ) in the remodeling plexi of *Gja4* ^−/−^ and WT+DAPT retinas (Fig. 4b, graph). Finally, when *Gja4* ^−/−^ mutants were crossed with *Cdt1*-mOrange+ reporter mice, in which cells in G1 phase of the cell cycle express CDT1-mOrange fusion protein, *Gja4* ^−/−^ remodeling retinal vascular plexi had fewer endothelial cells in G1 phase, consistent with dysregulation of cell cycle arrest (Fig. 4c).

Next, we assessed whether GJA4 was responsible for the effects of NOTCH on endothelial cell cycle status. We transfected HUVEC with full-length mouse *Gja4* (lenti-*Gja4* ^33^) to constitutively express GJA4 (or with lenti-Ctrl for control cells). As previously described^33^, lenti-*Gja4* effectively induced normal levels of GJA4 protein, which properly localized to cell-cell junctions (Supplementary Fig. 12A). Cells were then treated for 24 h with 10 µM DAPT, which we previously showed effectively blocks NOTCH signaling (Figs. 1, 2 and Supplementary Fig. 6), and cell cycle distribution was assessed. Control (DMSO-treated) cells had the following cell cycle distribution: 27.8 ± 4.9% in G0, 52.5 ± 3.8% in G1 and 19.6 ± 2.1% in S/G2/M. Compared to controls, DAPT-treated HUVEC, in which GJA4 expression is significantly reduced (Fig. 2d), exhibited a decreased proportion of cells in G1, and an increased proportion of actively cells in S/G2/M. However, constitutive re-expression of GJA4 abolished these DAPT-mediated cell cycle effects (Fig. 4d), indicating that NOTCH regulates cell cycle status via GJA4.

### GJA4 regulates cell cycle status via CDKN1B

Cell cycle progression is tightly regulated by cyclins and associated regulatory proteins. To identify cell cycle regulators targeted by FSS-activated NOTCH and GJA4, we examined our whole RNA-Seq data sets to determine whether the expression of cell cycle effectors were altered by FSS, and whether that expression was further affected by DAPT. We found that several cell cycle regulators were altered by 6 h FSS (vs. 6 h Static) (Supplementary Fig. 13A), including the cyclin-dependent kinase inhibitor *CDKN1B*, which encodes for CDKN1B (commonly, p27), an inhibitor of G1-S transition. CDKN1B expression was upregulated by FSS, and was also significantly downregulated by DAPT under FSS (Supplementary Fig. 13B), indicating that shear-induced CDKN1B was dependent upon NOTCH signaling.

**Fig. 5:**
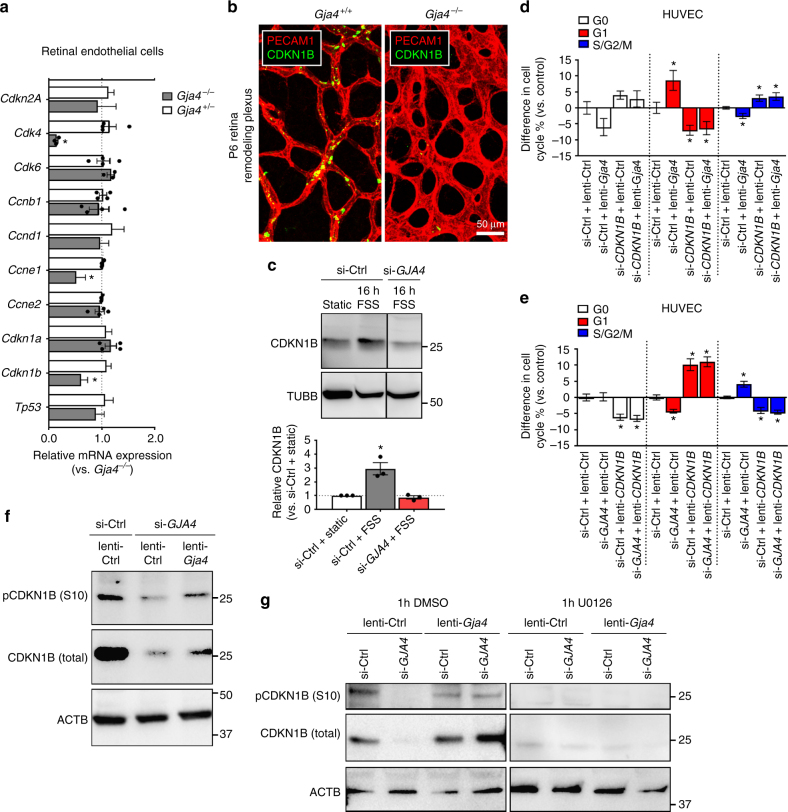
GJA4 regulates endothelial cell cycle arrest via CDKN1B. **a** *CDKN1B* mRNA expression was significantly reduced in *Gja4* ^−/−^ P6 retinal endothelial cells (CD31+/CD45−) (mean relative mRNA expression ± SEM vs. *Gja4* ^+/−^ littermate controls; *n* = 3 (*Cdk4*, *Gja4* ^−/−^; *Cdk6*, *Gja4* ^−/−^ *Ccne1*, *Gja4* ^+/−^; *Ccne2*, *Gja4* ^+/−^), *n* = 4 (*Cdk4*, *Gja4* ^+/−^; *Cdk6*, *Gja4* ^+/−^; *Ccnb1*; *Cdkn1a*, *Gja4* ^−/−^), *n *= 5 (*Ccne1*, *Gja4* ^−/−^), *n* = 6 (*Ccne2*, *Gja4* ^−/−^; *Cdkn1a*, *Gja4* ^+/−^; *Ccnd1*, *Gja4* ^−/−^), *n* = 8 (*Cdk2*; *Tp53*, *Gja4* ^−/−^), *n* = 9 (*Ccnd1*, *Gja4* ^+/−^; *Cdkn1b*; *Tp53*, *Gja4* ^−/−^); individual values plotted where *n* < 5). **b** CDKN1B expression was detected in *Gja4* ^+/+^, but not *Gja4* ^−/−^, endothelial cells of remodeling retinal vessels. (Colors: PECAM1 (red), CDKN1B (green); scale bar: 50 µm). **c** CDKN1B protein was elevated in HUVEC by 16 h FSS, and this effect was abolished by si-*GJA4* (mean densitometry ± SEM vs. Static; *n* = 3 for all groups; one-way ANOVA: *p* = 0.002, asterisks indicate *p *< 0.05 in post hoc *t*-test). Uncropped western blots presented in Supplementary Fig. 14A. **d** Constitutive GJA4 expression (lenti-*Gja4*) arrested endothelial cells in G1 and reduced actively cycling cells in S/G2/M, while si-*CDKN1B* had the opposite effect, regardless of GJA4 expression (mean difference in cell cycle % ± SEM vs. Control; *n* = 6; one-way ANOVA: *p* = 0.0002 (G1), *p* = 0.0003 (S/G2/M), asterisks indicate *p* < 0.05 in post hoc *t*-test vs. si-Ctrl + lenti-Ctrl). **e** GJA4 knockdown (si-*GJA4*) reduced the proportion of endothelial cells in G1 and increased the proportion in S/G2/M, and constitutive expression of CDKN1B (lenti-*CDKN1B*) had the opposite effect, regardless of GJA4 expression (mean difference in cell cycle % ± SEM vs. Control; *n *= 3 for all groups; one-way ANOVA: *p* = 0.002 (G0), *p* < 0.0001 (G1), *p* < 0.0001 (S/G2/M), asterisks indicate *p* < 0.05 in post hoc *t*-tests vs. si-Ctrl+ lenti-Ctrl). **f** CDKN1B phosphorylation at serine 10 (pCDKN1B (S10)) and total protein levels were reduced by si-*GJA4* and rescued with lenti-*Gja4*. Uncropped blots presented in Supplementary Fig. 14B. **g** MAPK/ERK signaling inhibition by 1 h treatment with 20 µM U0126, an inhibitor of MEK1/2, blocked GJA4-dependent CDKN1B phosphorylation at serine 10 and reduced total CDKN1B protein levels. Uncropped blots presented in Supplementary Fig. 14C

To determine whether cell cycle effectors identified as both FSS-activated and NOTCH-dependent were also regulated by GJA4, we next assessed expression of candidate cell cycle effectors in sorted *Gja4* ^−/−^ retinal endothelial cells (which we defined as events positive for PECAM1 (or, CD31) and negative for PTPRC (or, CD45)). Expression of CDKN1B (as well as its regulatory targets CDK4 and CCNE1) was significantly reduced in *Gja4* ^−/−^ retinal endothelial cells compared to *Gja4* ^+/−^ littermate controls (Fig. 5a); we detected no differences in the expression of other cycle effectors assessed. In WT (*Gja4* ^+/+^) P6 mice, CDKN1B was expressed in PECAM1-positive vessels of the remodeling plexus, but not detected in the remodeling plexus of *Gja4* ^−/−^ P6 mice (Fig. 5b). Furthermore, FSS-induced upregulation of CDKN1B protein expression was abrogated in HUVEC when GJA4 expression was suppressed by si-*GJA4* knockdown (Fig. 5c and Supplementary Fig. 14A).

We next investigated whether CDKN1B was necessary for GJA4 effects on endothelial cell cycle status. Control HUVEC treated with empty lentivirus (lenti-Ctrl) and control siRNA (si-Ctrl) had the following cell cycle distribution: 38.9 ± 0.02% in G0, 44.6 ± 0.02% in G1, and 16.9 ± 0.004% in S/G2/M. Constitutive expression of GJA4 (via lenti-*Gja4*) increased the proportion of cells in G1 and decreased the proportion of cells in S/G2/M (Fig. 5d). In contrast, siRNA knockdown of CDKN1B (si-*CDKN1B*, validated in Supplementary Fig. 12B, C) increased the proportion of actively cycling cells in S/G2/M and decreased cells in G1, regardless of GJA4 expression (Fig. 5d).

Similarly, HUVEC were transfected with si-*GJA4* prior to lentiviral delivery of human CDKN1B (lenti-*CDKN1B*, validated in Supplementary Fig. 12D, E). The cell cycle distribution of HUVEC transfected with si-Ctrl and infected with lenti-Ctrl was like that of other control groups: 35.5 ± 1.3% in G0, 48.6 ± 1.0% in G1, and 15.6 ± 0.5% in S/G2/M (Fig. 5e). GJA4 knockdown (via si-*GJA4*) decreased the proportion of cells in G1 and increased the S/G2/M fraction (Fig. 5e). Constitutive expression of CDKN1B, whether in the presence or absence of GJA4, had the opposite effect, increasing the proportion of cells arrested in G0/G1 and decreasing the proportion of actively cycling S/G2/M cells (Fig. 5e). Therefore, GJA4 regulates G1 arrest of endothelial cells via CDKN1B.

### MAPK/ERK signaling is required for GJA4 regulation of CDKN1B

Although CDKN1B is transcriptionally regulated, CDKN1B protein intracellular localization and function are primarily influenced by its phosphorylation on serine and threonine residues^34^. A significant site of CDKN1B phosphorylation is serine 10 (S10)^35^, which regulates nuclear export^36^ and protein stability^35,37^. In cycling cells, serine 10 phosphorylation is associated with CDKN1B ubiquitination and degradation to promote G1/S transition, whereas in quiescent cells, phosphorylation at serine 10 enhances protein stability to limit cell cycle progression^37,38^. We found that serine 10 phosphorylation (pCDKN1B (S10)) was reduced after GJA4 knockdown (via si-*GJA4*) in association with reduced total CDKN1B protein levels, which was rescued by re-expression of GJA4 (Fig. 5f and Supplementary Fig. 14B). These data suggest that GJA4 regulates CDKN1B stability and function and that this may involve serine 10 phosphorylation of CDKN1B protein.

Previous studies have shown that CDKN1B serine 10 phosphorylation is mediated by members of the MAPK family^35^. The GJA4 C-terminus contains a high-probability MAPK target consensus sequence^39^, and C-terminal truncation eliminates GJA4 suppression of cancer cell proliferation^40^, suggesting involvement of this site. To assess whether MAPK/ERK signaling is required for Cx37-mediated phosphorylation and stabilization of CDKN1B, we treated HUVEC for 1 h with 20 µM U0126, a specific inhibitor of MEK1/2, which activates MAPK/ERK signaling. U0126 blocked the capacity of re-expressed GJA4 to preserve serine 10 phosphorylation and total CDKN1B protein levels (Fig. 5g and Supplementary Fig. 14C). These results demonstrate that GJA4 requires MAPK/ERK signaling to stabilize CDKN1B protein.

### Arterial development requires GJA4 and NOTCH

**Fig. 6:**
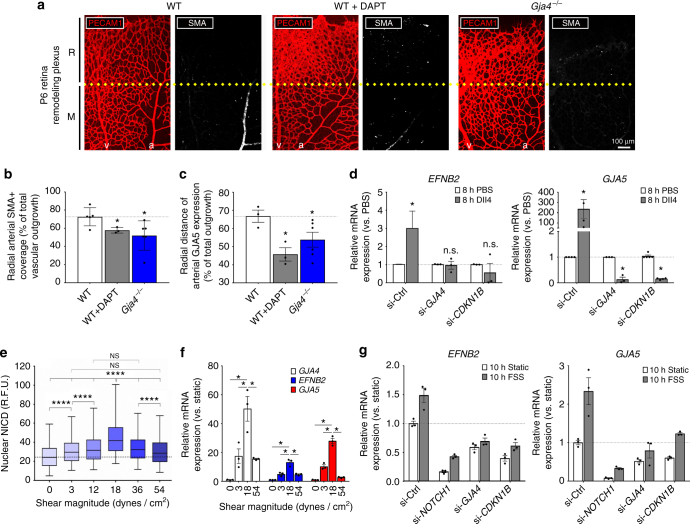
FSS-NOTCH-GJA4-CDKN1B axis regulates arterial identity genes. **a** Disorganized or absent SMA investment was observed in *Gja4* ^−/−^ and WT + DAPT mice, compared to WT controls. (Colors: PECAM1 (red), SMA (white); scale bar: 100 µm; symbols: a = artery, v = vein, R = remodeling, M = mature). **b** Radial SMA investment of arteries, as well as **c** radial distance of GJA5 (Cx40) expression, were reduced in *Gja4* ^−/−^ and DAPT-treated animals (mean radial distance as % of total vascular outgrowth ± SEM vs WT; *n* = 3 (WT + DAPT; WT, GJA5), *n* = 4 (WT, SMA), *n* = 6 (*Gja4* ^−/−^); one-way ANOVA: *p* = 0.03 (SMA), *p* = 0.04 (GJA5); asterisks indicate *p *< 0.05 in post hoc *t*-tests vs. WT). **d** DLL4 activation of NOTCH signaling upregulated *EFNB2* (EphrinB2) and *GJA5*, and this effect was abolished with si-*GJA4* or si-*CDKN1B* (mean relative mRNA expression ± SEM vs. PBS; *n* = 3 (si-*GJA4*; si-*CDKN1B, EFNB2*), *n* = 4 (si-Ctrl, *GJA5*; si-*CDKN1B*, *GJA5*), *n* = 4 (si-Ctrl, *EFNB2*); Students’ *t*-test: *p* = 0.02 (si-Ctrl, *EFNB2*), *p* = 0.04 (si-Ctrl, *GJA5*), *p* = 0.0005 (si-*GJA4*, *GJA5*), *p* < 0.0001 (si-*CDKN1B*, *GJA5*)). **e** NOTCH activation was maximal with 1 h exposure to 18 dynes/cm^2^ shear (mean NICD R.F.U. ± SEM; *n* = 7000 cells, representative of *N* = 3). **f** Expression of *GJA4* (Cx37), *GJA5* (Cx40) and *EFNB2* (EphrinB2) were also maximal at 18 dynes/cm^2^ (mean relative mRNA expression ± SE; *n* = 3, representative of 4 experiments). **g** Knockdown of NOTCH1, GJA4 or CDKN1B reduced basal and 10 h FSS-induced upregulation of *EFNB2* and *GJA5* (mean relative mRNA expression ± SEM vs. Static; *n* = 3 technical replicates; representative of two biological replicates)

To determine the effects of remodeling plexus hyperproliferation (Fig. 4) on vascular maturation, we assessed mural cell investment in maturing arteries. In WT tissue, SMA labeling extends from the center of the retina to the R-M boundary (Fig. 6a). By contrast, SMA staining was markedly reduced or absent in DAPT-treated WT retinas, as well as in *Gja4* ^−/−^ retinas. Quantification of these images showed ~ 20% decrease in the radial distance of SMA-positive coverage in NOTCH-inhibited and *Gja4* ^−/−^ animals (Fig. 6b), suggesting delayed arterial development. We further observed a significant decrease in the radial distance of GJA5 (commonly, Cx40) expression along the arteries of DAPT-treated WT and *Gja4* ^−/−^ tissues (Fig. 6c), consistent with a defect in acquisition of endothelial arterial identity.

### FSS-induced NOTCH-GJA4 signaling regulates arterial identity

In our RNA-seq studies, we found that 6 h of FSS significantly increased the expression of arterial identity genes *GJA5* and *EFNB2* (commonly, EphrinB2). We next explored whether GJA4 and CDKN1B function downstream of FSS-induced NOTCH signaling to promote arterial identity. We found that activation of NOTCH by DLL4 increased expression of *EFNB2* (EphrinB2) (and *GJA5*) (Fig. 6d). However, knockdown of either GJA4 (via si-*GJA4*) or CDKN1B (via si-*CDKN1B*) prevented DLL4-induced upregulation of *EFNB2*, and significantly reduced *GJA5* expression (Fig. 6d), indicating that GJA4 and CDKN1B function downstream of NOTCH signaling to enable arterial gene expression.

However, endothelial cells must be sensitive to the magnitude of flow during vascular morphogenesis in order to optimize arterial-venous specification and attain proper tissue perfusion^3^. To test this, endothelial cell monolayers were exposed to 1 h of shear over a wide physiological range of FSS magnitudes (0–50 dynes/cm^2^). NOTCH cleavage (Fig. 6e), GJA4 expression, and expression of arterial genes *EFNB2* and *GJA5* (Fig. 6f) peaked at intermediate FSS magnitudes typical of arteries, and was reduced at magnitudes outside this range, demonstrating that arterial FSS activates NOTCH-GJA4 signaling to enable arterial specification.

Next, we used siRNA to knockdown NOTCH1, GJA4, and CDKN1B under arterial FSS magnitudes (18 dynes/cm^2^) to determine the contribution of NOTCH-GJA4-CDKN1B signaling to shear-induced upregulation of arterial genes. NOTCH1, GJA4 and CDKN1B knockdown did not affect shear-activated *KLF2* expression, indicating that mechanotransduction signaling complexes were intact (Supplementary Fig. 15). Basal and FSS-induced *EFNB2* and *GJA5* expression was reduced in all groups compared to control cells, and GJA4 knockdown (via si-*GJA4*) completely abolished FSS-induced upregulation of arterial identity genes (Fig. 6g).

### Endothelial G1 arrest is needed for arterial specification

**Fig. 7:**
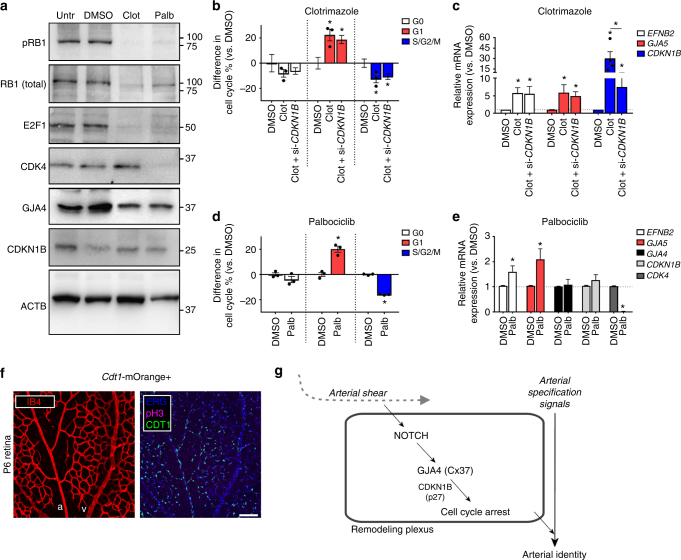
Endothelial cell cycle arrest, per se, enables arterial gene expression. **a** Treatment of HUVEC with 10 µM clotrimazole or 2 µM palbociclib reduced RB1, phosphorylated RB1 (pRb1), and E2F1, suggestive of G1 arrest. Clotrimazole tended to upregulate CDKN1B expression and preserve GJA4 expression, and CDK4 expression was lost only with CDK4/6i treatment. Uncropped blots presented in Supplementary Fig. 16. **b** Using FACS to assess cell cycle distribution, clotrimazole was found to arrest HUVEC in G1, even when CDKN1B was knocked down (via si-*CDKN1B*) (mean difference in cell cycle % ± SEM vs. DMSO; *n* = 3 (Clotrimazole), *n* = 5 (DMSO), *n* = 6 (Clotrimazole+ si-*CDKN1B*); individual values plotted where *n* < 5; one-way ANOVA: *p* = 0.007 (G1), *p* = 0.03 (S/G2/M); asterisks indicate *p *< 0.05 in post hoc *t*-tests). **c** Clotrimazole upregulated *EFNB2* and *GJA5* regardless of CDKN1B expression (mean relative mRNA expression ± SEM vs. DMSO; *n* = 4 (Clotrimazole, p27), n = 5 (Clotrimazole, *EFNB2*; Clotrimazole, *GJA5*), *n* = 7 (Clotrimazole+ si-CDKN1B), *n* = 8 (DMSO); individual values plotted where *n* < 5; one-way ANOVA: *p* = 0.03 (*EFNB2*, *GJA5*), *p* = 0.0007 (*CDKN1B*)). **d** Palbociclib treatment also arrested endothelial cells in G1 (mean difference in cell cycle % ± SEM vs. DMSO; *n* = 3 for all groups; Students’ *t*-test: *p* = 0.002 (G1), *p* < 0.0001 (S/G2/M)), **e** upregulated *EFNB2* and *GJA5* (Cx40) mRNA levels, abolished *CDK4* expression, and had no effect on *GJA4* (Cx37) and *CDKN1B* (p27), (mean relative mRNA expression ± SEM vs DMSO; n = 9 (*GJA4*), *n* = 12 (*EFNB2*), *n* = 14 (*GJA5*), *n* = 16 (*CDKN1B*; *CDK4*); Students’ *t*-test: *p* = 0.02 (*EFNB2*, *GJA5*), *p* < 0.0001 (*CDK4*)). **f** Endothelial cells within arteries and surrounding plexi of P6 retinas from *Cdt1*-mOrange+ reporter mice were predominantly in G1 phase, whereas endothelial cells in adjacent veins were not. (Colors: IB4 (red), ERG (blue), pH3 (magenta), CDT1 (green); scale bar: 100 µm). **g** We hypothesize that in remodeling vessels, arterial shear activates a novel Notch-Cx37-p27 signaling pathway that promotes endothelial cell cycle arrest to enable arterial gene expression

The above results prompted us to hypothesize that FSS-activated NOTCH-GJA4-CDKN1B signaling enables arterial specification via cell cycle arrest. However, as CDKN1B may also regulate transcription independent of cell cycle effects^34,41^, we tested whether cell cycle arrest, per se, mediates arterial gene expression. HUVEC were treated with 10 μM clotrimazole, which causes G1 arrest via a CKN1B-independent mechanism^42^ (although it modestly upregulates CDKN1B as a secondary effect), or with 2 µM palbociclib^43^, which specifically eliminates expression of CDK4/6 without affecting CDKN1B or GJA4 expression (Fig. 7a and Supplementary Fig. 16). Both drugs reduced total and phosphorylated levels of RB1, as well as E2F1, consistent with block of G1-to-S phase transition (Fig. 7a). Both drugs also increased the proportion of endothelial cells in G1, decreased endothelial cells in S/G2/M (Fig. 7b, d), and increased expression of arterial markers *EFNB2* and *GJA5* (Fig. 7c, e). Knockdown of CDKN1B had no effect on clotrimazole-induced G1 arrest (Fig. 7b) or on the upregulation of arterial genes (Fig. 7c). Collectively, these data indicate that G1 arrest enables arterial specification. Consistent with this model, evaluation of *Cdt1*-mOrange reporter mice revealed that endothelial cells in G1 are more abundant in arterial endothelial cells and surrounding plexi compared to veins (Fig. 7f).

Altogether, these data show that in remodeling endothelial cells, arterial shear activates a novel NOTCH-GJA4-CDKN1B (or, Notch-Cx37-p27) signaling pathway that promotes endothelial cell cycle arrest to enable arterial specification (Fig. 7g).

## Discussion

During blood vessel remodeling, endothelial cell growth suppression and arterial-venous specification occur simultaneously, but how these processes are regulated and coordinated was not known. Herein, we demonstrate that endothelial G1 cell cycle arrest is required for expression of arterial-specific genes. In addition, FSS, specifically in the range typical of arterioles and arteries, initiates growth arrest by activating NOTCH signaling, which promotes endothelial cell cycle arrest via GJA4 and downstream CDKN1B in remodeling vessels. These findings are consistent with the observation that NOTCH signaling is low in veins^44^, and that upregulation of venous endothelial gene expression is not observed upon exposure to pulsatile blood flow^4^.

NOTCH signaling was previously thought to promote arterial development primarily via upregulation of fate-specific genes (reviewed in Marcelo et al.^2^). However, our findings suggest a new paradigm in which the primary role of NOTCH signaling in arterial-venous specification is to modulate endothelial cell cycle state, similar to the role of NOTCH in hemogenic endothelial cell specification^16^. How endothelial cell cycle arrest in G1 leads to arterial specification is unknown, but studies in embryonic stem cells may offer a clue. Stem cells in different cell cycle phases respond differently to inductive signals^18^. For example, early G1 arrest enables responses to factors that promote mesodermal or endodermal vs. ectodermal commitment^45,46^. Arterial inductive signals may therefore be similarly confined to endothelial cells in G1. Chromatin structure, epigenetic regulation, and accumulation of transcription factors, cofactors, and miRNAs may all potentially contribute to cell cycle-dependent sensitivity to extrinsic cues.

The mechanism by which FSS at arterial magnitudes activates NOTCH is unknown. That NOTCH is acutely activated at 1 h following onset of shear makes transcriptional regulation unlikely. Furthermore, although NOTCH signaling is innately mechanosensitive^47^, the kinetics of NOTCH activation argue against direct activation of NOTCH by fluid shear forces. Instead, fluid shear stress-induced signal transduction could result in altered membrane-cytoskeletal rearrangements, as is observed within the CDH5 (VE-Cadherin)/PECAM1 (CD31) complex^48^ and mechanically induced NOTCH cleavage^47^. Alternatively, shear signaling could modify stabilization and/or membrane trafficking of NOTCH and its ligands. Finally, shear-induced alterations in membrane lipid composition might alter gamma-secretase complex activity to indirectly enhance NOTCH cleavage^49^. Thus, understanding activation of NOTCH by flow will require further investigation.

Also unknown is how GJA4, downstream of NOTCH, regulates CDKN1B phosphorylation and possibly MAPK/ERK signaling. In insulinoma cells, which typically do not express connexins, enforced expression of GJA4 suppresses proliferation^50^ in a manner that requires both gap junction channel function and the C-terminal region of the protein, which is dispensible for gap junction channel formation and instead mediates intracellular signaling^33,40^. Interestingly, truncation of the GJA4 C-terminus also eliminates a high-probability MAPK consensus site^39^ that may be required, either alone and/or in combination with other kinase target sites, for GJA4 to suppress cell growth. Further studies of GJA4 function and interactions in endothelial cells will be needed to address these questions.

Finally, although our studies were conducted in a developmental setting, the FSS-NOTCH-GJA4-CDKN1B (or FSS-Notch-Cx37-p27) pathway we characterize herein is likely also relevant to vascular remodeling in adults. Mice deficient in GJA4^20,32^ or CDKN1B^51^ display abnormal post-ischemic vessel growth and remodeling. Flow-induced Notch-Cx37-p27 endothelial cell cycle control is also likely to be important for maintenance and/or repair of adult vasculature. Thus, Notch-Cx37-p27 signaling may be an attractive candidate for treatment of vascular diseases, and for promotion of proper vessel formation in tissue engineering and regenerative medicine applications.

## Methods

### Cell culture

Primary human umbilical vein endothelial cells (HUVEC) were obtained from the Yale core facility and only used up to passage 8. The cells were cultured in M199 with 20% FBS (Gibco, Gemini), 100 µg/mL Heparin (Sigma), 50 µg/mL endothelial cell growth supplement (Sigma), and 1× penicillin/streptomycin (Sigma); some received 10 μM DAPT (Sigma) or DMSO, or were seeded onto surfaces pre-treated with 10 μg/mL recombinant human Dll4 (R&D). For siRNA knockdown, cells pre-conditioned with EGM-2 (Lonza) for >16 h were transfected with 20 nM si-*GJA4* (Dharmacon L-011669-00), si-*CDKN1B* (Dharmacon L-040178-00), si-*NOTCH1* (Dharmacon L-007771-00), si-*NOTCH4* (Dharmacon L-011883-00) or control siRNA (Ambion AM4611) for >96 h. For lentiviral transduction, HUVEC were pre-infected for 48 h with lentivirus containing mouse *Gja4* (lenti-*Gja4*)^33^, human CDKN1B (lenti-*CDKN1B*, cloned from Addgene 14049) or empty vector in presence of 10 μg/mL polybrene (Sigma). In MEK1/2 inhibitor studies, cells were washed with fresh EGM-2 media overnight, and then treated for 1 h with 20 µM U0126 (Sigma) or DMSO. In cell cycle studies, cells were treated with 24 h 10 μM clotrimazole (Sigma), 8 h 2 µM palbociclib (Sigma), or DMSO for the equivalent period. Cell cultures were routinely tested for mycoplasma contamination, and no positive test was obtained over the course of these studies.

### Immunochemistry reagents

For Western blot: α-hGJA4 (Abcam ab181701 1 : 3000; or, Santa Cruz sc-27711 1 : 1000), α-CDKN1B (Cell Signaling 3686 1 : 3000; or, Santa Cruz sc-527 1 : 1000), α-NOTCH-ICD (Cell Signaling 4147 s 1 : 3000), α-RB1 (Cell Signaling 9309 1 : 3000), α-phosphorylated RB1 (Cell Signaling 9308 1 : 3000), α-E2F1 (Cell Signaling 3742 1 : 3000), α-CDK4 (Cell Signaling 12790 1 : 3000), α-TUBB (Tubulin) (Sigma-Aldrich T9026 1 : 3000), α-ACTB (β-Actin) (Santa Cruz sc-1616, 1 : 500) and HRP-conjugated secondary antibodies (GE 1 : 5000). For immunocytochemistry: α-mGJA4 (Cx37) (Invitrogen 42-4500 1 : 300), α-mGJA5 (Cx40) (Invitrogen 36-4900 1 : 300), α-phospho-HIST3 (pH3) (Upstate 06-570 1 : 300; or, Sigma H6409 1 : 300), α-pERK5 (EMD Millipore 07-507 1 : 200), α-CDKN1B (NeoMarkers MS-256-P1 1 : 100) α-mPECAM1 (R&D AF3628 1 : 500), α-SMA (Dako M0851 1 : 300), α-ERG (Abcam ab92513 1 : 500), Alexa488-conjugated isolectin B4 (IB4, Life Technologies 121411 1 : 300), and fluorescently conjugated secondary antibodies (Life Technologies 1 : 1000).

### Shear

HUVEC were cultured on 25 × 55mm cell plastic slides pre-treated with 10 µg/mL fibronectin. Slides were then either maintained static in a cell culture incubator or mounted into parallel plate flow chambers and subjected to 3–50 dynes/cm^2^ laminar shear for up to 24 h^52^. Flow was applied using a pressure-dampened, gravity-driven dual reservoir system with media re-circulating via a peristaltic pump (Masterflex, Cole Palmer). Media was maintained at 37 C, 5% CO2 using a Digi-sense temperature control system (Cole Palmer) and humidified bubbler, respectively. Where indicated, cells were sheared in the presence of 10 µM DAPT. For EdU assays, 10 µM EdU was added for 2 h under shear prior to visualization (ThermoFisher Click-iT). Incorporation was quantified by imaging eight random fields at low magnification and averaging the frequency of EdU-positive nuclei vs. total nuclei. For some experiments, cleaved NOTCH receptor was detected by immunocytochemistry (Cell Signaling) following shear, and fluorescent intensity was quantified for ~6500 cells per treatment group using Matlab.

### RNA-Seq

HUVEC were exposed to shear (or static) conditions in triplicate, mRNA was isolated (Qiagen, RNeasy Plus Micro Kit), and cDNA libraries were prepared (ABM). Next-generation whole-transcriptome Illumina sequencing was performed with the Yale Center for Genome Analysis. RNA-Seq reads from each sample were aligned to human genome build 38 (GRCh38) using short reads aligner STAR^53^. Gene expression quantification was performed using RSEM^54^ with GENCODE annotation (release 24: http://www.gencodegenes.org). Differential analysis was performed using edgeR^55^ to identify genes with significant expression changes between groups (6 h Static vs. 6 h Shear; or 6 h Shear, Untreated vs. DAPT). Gene transcripts observed to change significantly (false discovery rate < 0.001) between static and shear groups were used for functional enrichment analysis by running GOseq^56^. A modified version of the nEASE algorithm^57,58^ was used to assess functional enrichment of nested GOseq (nGO) terms. Briefly, each enriched upper-level GOseq (GO) term was then used for nGO analysis to identify statistically enriched nGO terms driving upper-level functional enrichment of non-specific GO terms. GO-nGO paired terms relating to cell proliferation, cell signaling and development were selected for further consideration (Supplementary Data 1). Genes in Supplementary Data 1 were searched against established Kegg signaling modules (http://www.kegg.jp/kegg/tool/map_module.html); modules lacking upper-level block hits were discarded to yield relevant flow-sensitive signaling pathways.

### Quantitative PCR

Transcriptomes were prepared from cultured HUVEC or PECAM1+/PTPRC− (commonly, CD31+/CD45–) endothelial cells sorted from digested retinas of *Gja4* ^−/−^ neonates (or *Gja4* ^+/^^−^ littermates) using flow cytometry (see below, typical yield: ~2000 cells). Resulting cDNA libraries were probed by quantitative PCR (qPCR) using published qPCR primers (PrimerBank, sequences listed in Supplementary Table 4).

### Consensus sequence scanning

The mouse genomic promoter and coding sequence for *Gja4*—defined as −5 kb to +3 kb from the transcriptional start site of mouse *Gja4* mRNA sequence (Genbank #NM_008120)—was identified from published C57Bl/6 J GRCm38.p3 chromosome 4 assembly (positions 127311039–127319039, Genbank #NC_00070.6). The human genomic promoter and coding sequence for *GJA4* was also identified (positions 34789196–34797196 of GrCh38.p2 chromosome 1, with Genbank #NM_002060.2 as reference). Both sequences were scanned with DMINDA software^59^ to identify high-probability (*p* < 0.001) matches to the RBP-Jκ consensus motif (C/T)(C/G)TG(G/A)GA(A/G/T)^60,61^.

### ChIP

Chromatin-immunoprecipitation studies were performed according to manufacturer’s specifications (R&D ECP001) using α-RBP-Jκ (Santa Cruz sc-28713X 2.5 µg/mL) (or equivalent concentration of IgG control, Santa Cruz sc-2027X), biotinylated α-Rabbit (Vector BA-100 2.5 µg/mL) and streptavidin-conjugated agarose beads (Sigma 85881-1 mL). Resulting samples were probed with primers (Supplementary Table 4) targeting sequence flanking the human *GJA4* transcriptional start site.

### Animals

All animals were on a C57Bl/6 background, and all studies were conducted in compliance with Yale’s Institutional Animal Care and Usage Committee guidelines. Male and female *Gja4* ^−/−^ animals^62^ were compared to wild-type *Gja4* ^+/+^ or *Gja4* ^+/−^ littermates or age-matched wild-type C57Bl/6 animals (Jackson #025697), as indicated. *Gja4* ^−/−^ were also crossed with mice bearing a *Cdt1*-mOrange fusion protein^54^ (gift of S. Guo) to generate *Cdt1*-mOrange+;*Gja4* ^−/−^ mice (or *Cdt1*-mOrange+ ;*Gja4* ^+/+^ littermate controls). Some wild-type animals received two subcutaneous injections of 100 mg/kg DAPT (or drug vehicle) at 24 h and 12 h prior to killing; in these studies, littermates were randomized with regard to whether they receive drug or vehicle control. *Notch1* ^*iECKO*^ animals were generated by crossing *Notch1* ^*flox/flox*^ (Jackson #006951) mice with *Cdh5CreERt2* mice, which express a tamoxifen-inducible Cre recombinase in endothelial cells driven by a CDH5 (VE-Cadherin) promoter. NOTCH1 was post-natally deleted in endothelial cells by injection of 100 mg/kg tamoxifen (Sigma) at P1-3, prior to animal killing at P6; Cre+ NOTCH-deleted animals were compared against Cre− littermate controls.

### Intracardiac injection

Freshly killed P6 animals received a 500 μL bolus of 10 mg/mL lysine-fixable fluorescently conjugated 70 kDa rhodamine dextran (ThermoFisher) into the left ventricle. Eyes were removed and fixed in 4% formaldeyhyde for 18 min before retinal dissection and immunohistochemistry, as described above.

### Retinal vascular analysis

Neonatal mouse retinas (P3-21) were collected and stained as previously described^12,25–27^. In brief, eyes were pre-fixed in 4% paraformaldehyde and dissected retinas were post-fixed for 5 min in 4% paraformaldehyde at room temperature or for 20 min in 100% methanol at −20 °C. Retinas were incubated in blocking solution (3% BSA, 0.25% Triton-X in PBS) and incubated overnight in staining solution (1% BSA, 0.25% Triton-X in PBS) containing primary antibodies (listed above). Following PBS wash, retinas were incubated for 1 h at room temperature in staining solution containing secondary antibody (listed above); if staining with fluorescently conjugated IB4, staining solution is supplemented with 100 mg/mL CaCl_2_ and MgCl_2_. Retinas were then washed and mounted on glass slides for confocal imaging. The remodeling region was delineated as the distance between the vascular edge and the point where SMA signal is indistinguishable from background (ImageJ). GJA5 and SMA expression was calculated as the radial distance from the retinal center to the point at which fluorescent signal for each marker was undetectable. From a 769μm^2^ × 923μm^2^ ROI of the remodeling region, total vascular (i.e., PECAM1+ or IB4+) area (ImageJ) and plexus (Retina Analyser v.2, Centre de Morphologie Mathematique, École Nationale Superieure des Mines de Paris) and arterial (ImageJ) branchpoint number were calculated. Linear distance from vascular edge was measured in whole retinal quadrants for mitotic endothelial cells (pH3+/ERG+). Endothelial cells in G1 were identified in *Cdt1*-mOrange+ reporter mice as CDT1+/ERG+ cells. For all studies involving retinal vascular morphometric analysis, the investigator was blinded to animal genotype until imaging processing was complete.

### Flow cytometry

Age-matched *Gja4* ^−/−^ and *Gja4* ^+/+^ retinas were freshly dissected and digested in 1 mg/mL collagenase type II for 20 min in DMEM (Gibco) with 20% FBS (Gibco or Gemini) to obtain single-cell suspensions. Alternatively, HUVEC were lifted using 0.25% trypsin and resuspended in PBS. Cells were resuspended in 100 μL staining medium (HBSS with 10% FBS, 20 mM HEPES and 1 mg/mL d-glucose), and incubated for 15 min in 10 μg/mL Hoechst 33342 (Sigma) at 37 °C, and for 15 min with 0.5 μg/mL Pyronin Y (Sigma P9172) and/or fluorescently conjugated antibodies (α-mPECAM1-APC, BD Pharmingen 551262 1 : 100; α-mPTPRC-FITC, BD Pharmingen 553088 1 : 100). Washed pellets were resuspended in 0.1% FBS in PBS. Endothelial cells were identified as PECAM1+/PTPRC− (commonly, CD31+/CD45–) events and cell cycle analysis was performed by comparison of DNA (Hoechst) and RNA (Pyronin Y) content for each event.

### Statistics

Power analysis was performed on retinal vascular morphometry data collected from WT + DAPT (vs. WT + Vehicle) P6 tissue, since NOTCH inhibition is known to induce a hyperdensity phenotype in P6 retinas. Power analysis was also performed on cell cycle data from sorted retinal endothelial cells from WT + DAPT (vs. WT + Vehicle) to assess the statistical effect size of cell cycle distribution changes associated with vascular hyperdensity. Power analysis was also performed on HUVEC treated with 2 µM palbociclib (vs. DMSO), which has been shown to induce G1 arrest. Finally, power analysis was performed on *HES1* mRNA transcript levels in HUVEC treated with 10 µM DAPT (vs. DMSO) or 10 μg/mL DLL4 (vs. PBS), which is known to impact transcription of canonical NOTCH targets. In all cases, a minimum sample size of 3 was shown to be sufficient to detect significant differences at a power of 0.80 and *α* = 0.05, although pilot data also suggested that a sample size of 5 or greater was preferable. Therefore, for all experiments, we constrained sample size to a minimum of 3–5 with a maximum sampling ratio of 4. Analysis of data obtained through these pilot studies revealed that data were normally distributed and that variance was similar between sample and control groups.

For animal studies, in rare cases, pups were a priori discarded if they appeared to be underweight or lethargic at the time of study onset, but no animals were post hoc excluded from the data set. In rare cases, single data points obtained by qPCR were considered outliers and excluded from subsequent analysis if they fell >2 SD from the sample mean.

Unless otherwise indicated, statistics (*α* = 0.05) were performed using either a standard two-tail Student’s *t*-test, or a two-way ANOVA test, followed by post hoc *t*-test with Bonferroni correction.

### Code availability

Matlab code and other relevant data associated with these studies are available upon reasonable.

### Data availability

The RNA-Seq data sets generated and analyzed in the current study are available at the NCBI Sequence Read Archive (Accession Number: #SRP113256).

## Electronic supplementary material

Supplementary Information

Description of Additional Supplementary Files

Supplemental Data 1
