# Addendum: Shear-induced Notch-Cx37-p27 axis arrests endothelial cell cycle to enable arterial specification

**DOI:** 10.1038/s41467-018-03076-4

**Published:** 2018-02-14

**Authors:** Jennifer S. Fang, Brian G. Coon, Noelle Gillis, Zehua Chen, Jingyao Qiu, Thomas W. Chittenden, Janis M. Burt, Martin A. Schwartz, Karen K. Hirschi

**Affiliations:** 1 Department of Medicine, Yale University School of Medicine, 333 Cedar Street, New Haven, CT 06520 USA; 2 Yale Cardiovascular Research Center, Yale University School of Medicine, 333 Cedar Street, New Haven, CT 06520 USA; 3 Vascular Biology and Therapeutics Program, Yale University School of Medicine, 333 Cedar Street, New Haven, CT 06520 USA; 4 Yale Stem Cell Center, Yale University School of Medicine, 333 Cedar Street, New Haven, CT 06520 USA; 5 Computational Statistics and Bioinformatics Group, Advanced Artificial Intelligence Research Laboratory, WuXi NextCODE 55 Cambridge Parkway, 8th Floor, Cambridge, MA 02142 USA; 6 Department of Genetics, Yale University School of Medicine, 333 Cedar Street, New Haven, CT 06520 USA; 7 Division of Genetics and Genomics, Boston Children’s Hospital, Harvard Medical School, A-111, 25 Shattuck Street, Boston, MA 02115 USA; 8 Department of Biological Engineering, Massachusetts Institute of Technology, 21 Ames Street #56-651, Cambridge, MA 02142 USA; 9 Department of Physiology, College of Medicine, The University of Arizona, 1501 N. Campbell Road, Tucson, AZ 85724 USA; 10 Department of Cell Biology, Yale University School of Medicine, 333 Cedar Street, New Haven, CT 06520 USA; 11 Department of Biomedical Engineering, Yale University School of Medicine, 333 Cedar Street, New Haven, CT 06520 USA

**Keywords:** Angiogenesis, Differentiation

Addendum to: *Nature Communications* 10.1038/s41467-017-01742-7, published online 15 December 2017

Publication of this Article was delayed due to technical issues during the production process. The Article should have been co-published with a related paper by Iruela-Arispe and colleagues^1^.
