# Supplementary material for: Shear-induced Notch-Cx37-p27 axis arrests endothelial cell cycle to enable arterial specification
Source: Nat Commun. 2017 Dec 15;8:2149. doi: 10.1038/s41467-017-01742-7 (PMC5732288; doi:10.1038/s41467-017-01742-7)
Supplement: Supplementary file 1 — Supplementary Information [file 41467_2017_1742_MOESM1_ESM.pdf]

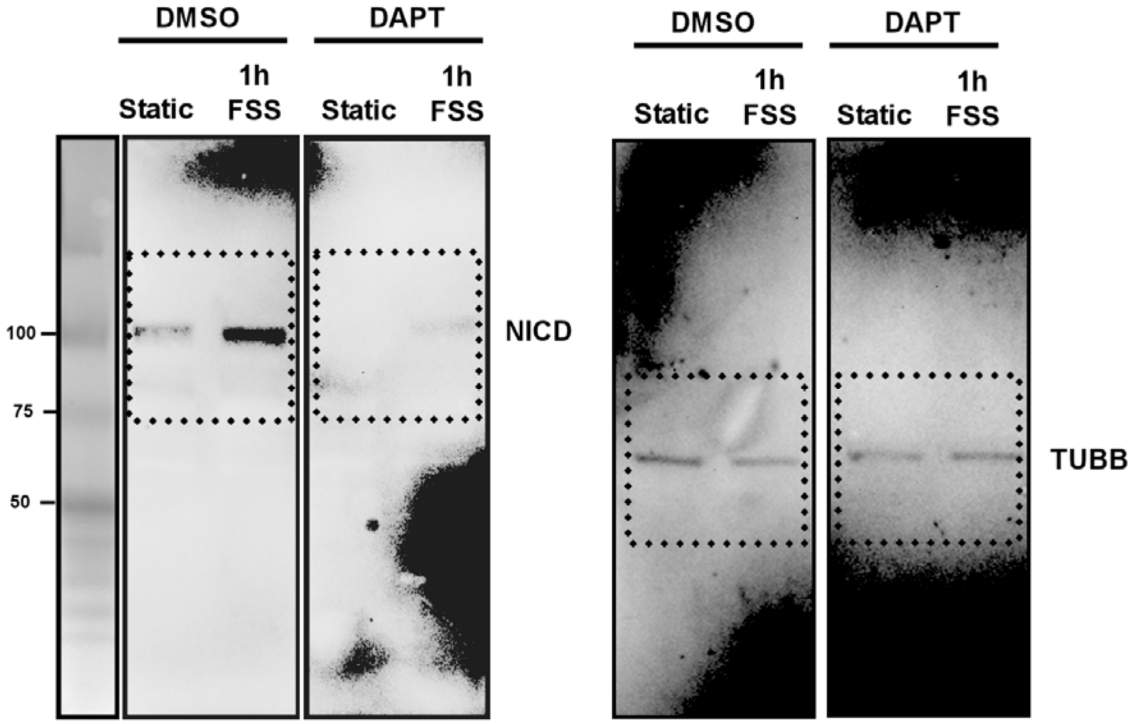

Supplementary Figure 1: Uncropped western blots for Figure 1B. Uncropped blots shown in **Figure 1B**, showing that NOTCH intracellular domain (NICD) is increased with exposure of HUVEC to 1h FSS, and that this increase is lost with exposure of cells to DAPT, an inhibitor of NOTCH receptor cleavage. Dotted lines indicate cropping for presentation in **Figure 1B**.

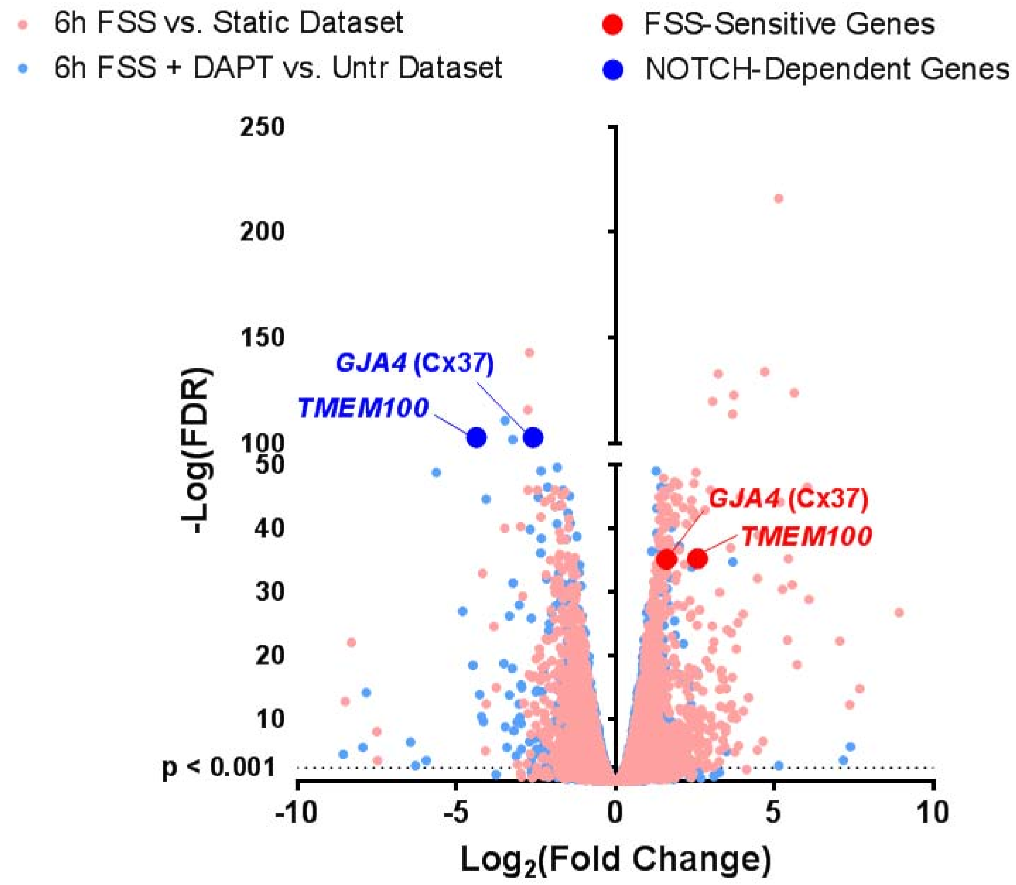

Supplementary Figure 2: *GJA4* and *TMEM100* are likely regulated by shear-activated Notch signaling. In RNASeq analysis, *GJA4* and *TMEM100* expression was significantly upregulated by 6h FSS, and significantly downregulated by addition of DAPT under 6h shear to inhibit Notch signaling.

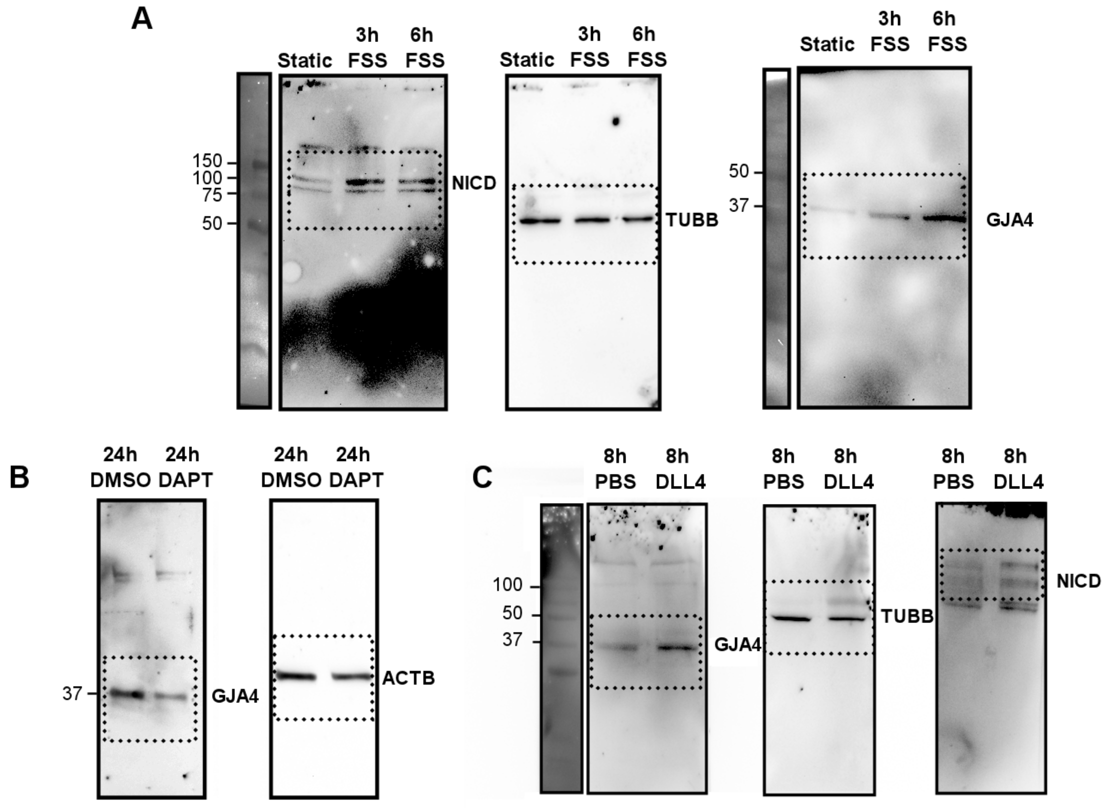

Supplementary Figure 3: Uncropped western blots for Figure 2. A) Uncropped western blots showing that FSS upregulates NICD and GJA4. **B)** Uncropped blots showing that 24h DAPT decreases GJA4 expression in confluent HUVEC. **C)** Uncropped western blots showing that 8h plating on immobilized recombinant DLL4 increases NICD and GJA4 expression. Dotted lines indicate cropping for presentation in **Figure 2**.

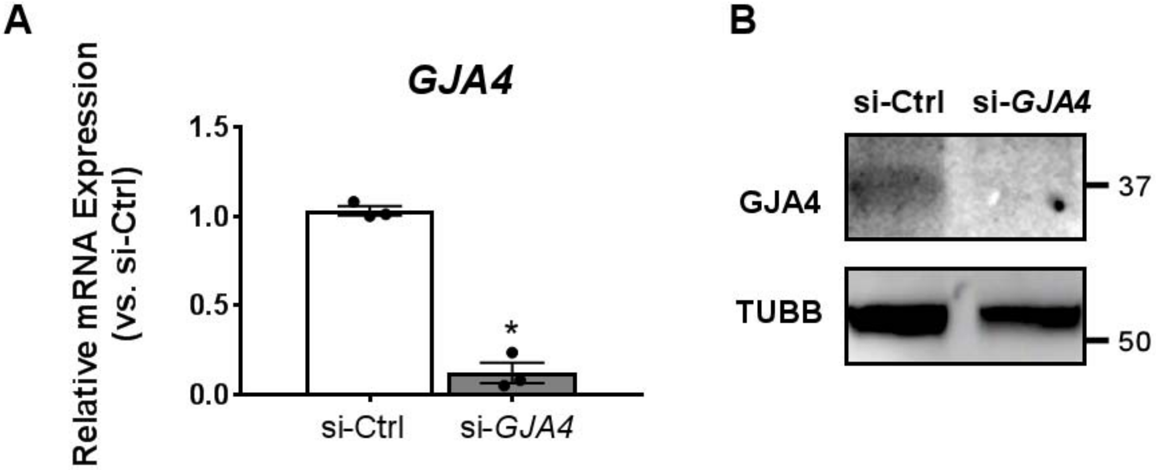

Supplementary Figure 4: si-*GJA4* treatment knocks down GJA4 mRNA and protein. **A)** Following 120h of exposure to si-*GJA4*, endogenous human *GJA4* mRNA was decreased by >95% compared to control HUVEC treated with scrambled (si-Ctrl) siRNA sequence (mean relative mRNA expression ± SEM; n=3 for all groups; Students' t-test: p=0.0001). **B)** Endogenous human GJA4 protein expression was observed in confluent si-Ctrl cells, but not detectible with si-*GJA4*.

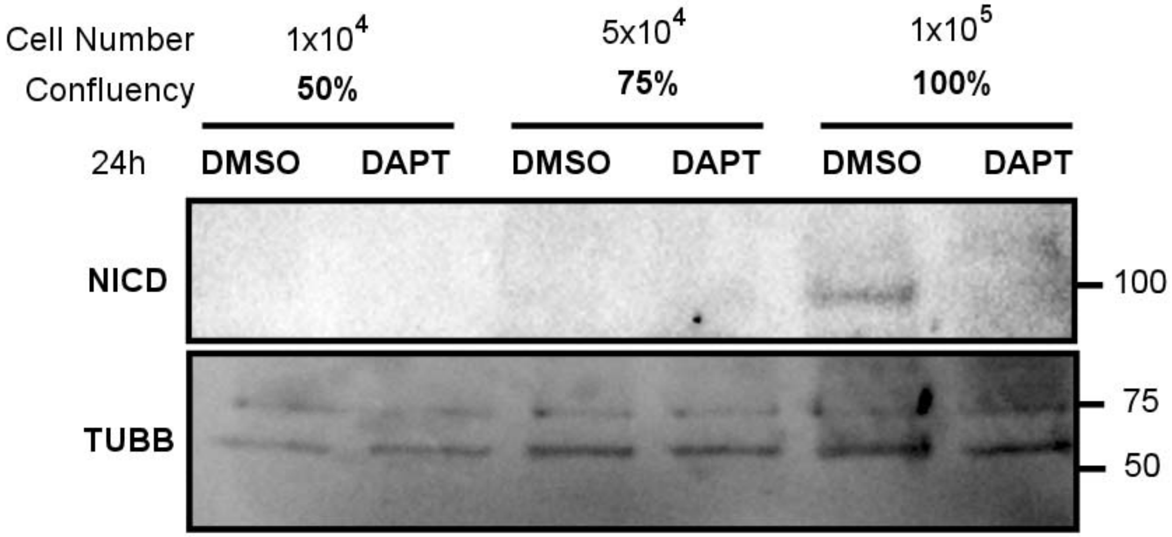

Supplementary Figure 5: Basal NOTCH signaling is robust in confluent HUVEC. Endogenous cleavage of the NOTCH receptor was observed in confluent, but not subconfluent HUVEC and was blocked with 10µM DAPT.

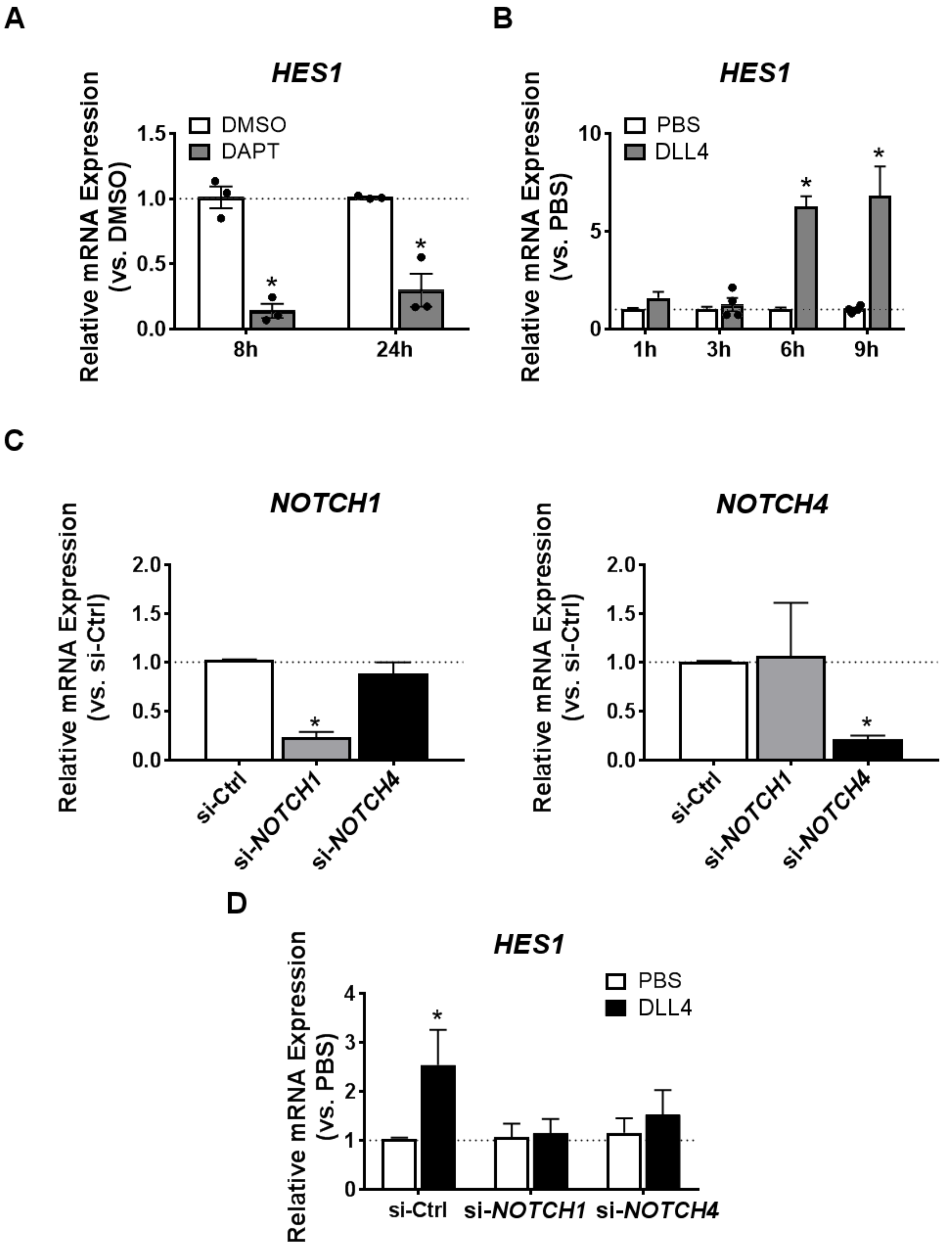

Supplementary Figure 6: NOTCH signaling altered by DLL4, DAPT and si-RNA. A) Expression of *HES1* mRNA was inhibited in confluent HUVEC with 6h and 24h exposure to 10μM DAPT (mean relative mRNA expression ± SEM vs. DMSO; n=3 for all groups; Students' t-test: p=0.0009 (8h), p=0.005 (24h)). **B)** *HES1* mRNA was upregulated at 6h

and 9h following seeding of subconfluent HUVEC on recombinant Dll4 (mean relative mRNA expression ± SEM vs. PBS; n=4 (9h PBS; 3h Dll4), n=5 (all other groups); individual values plotted for n<5; Students' t-test: p<0.0001 (6h), p=0.01 (9h)). **C)** Compared to si-Ctrl HUVEC, si-*NOTCH1* significantly reduced *NOTCH1* mRNA expression while si-*NOTCH4* significantly reduced *NOTCH4* expression. In either si-RNA treatment group, the non-targeted NOTCH receptor appeared to be unaffected (mean relative mRNA expression ± SEM vs. PBS; n=7 (si-Notch1), n=8 (si-Ctrl; si-*NOTCH4*); one-way ANOVA: p<0.0001 (*NOTCH1*), p=0.13 (*NOTCH4*), asterisks indicate p<0.05 in post-hoc t-test). **D)** Upregulation of *HES1* mRNA by seeding of subconfluent HUVEC on recombinant DLL4 for 6h was lost with si-*NOTCH1* or si-*NOTCH4* (mean relative mRNA expression ± SEM vs. PBS; n=7 (si-Notch1), n=8 (si-Ctrl; si-*NOTCH4*); Students' t-test: p=0.04 (si-Ctrl)).

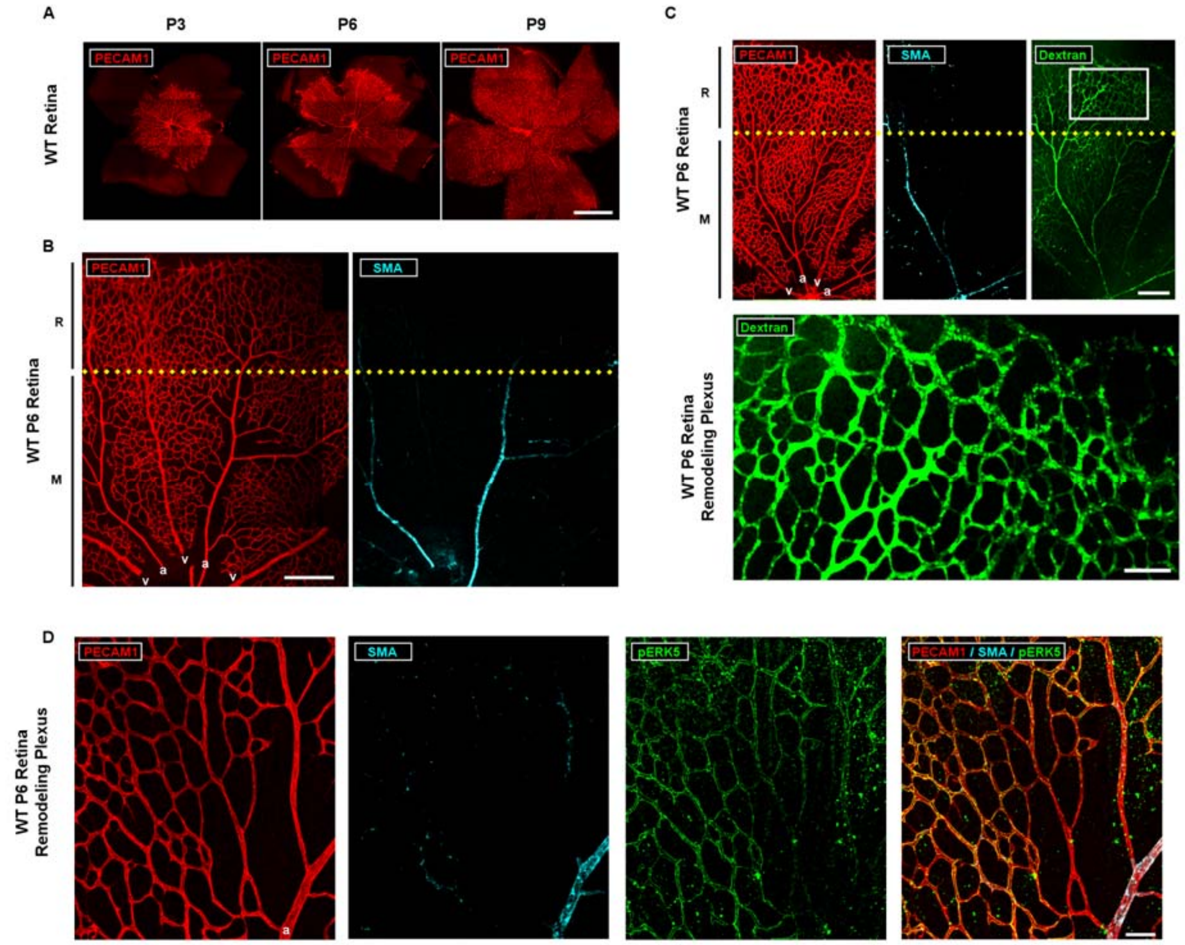

Supplementary Figure 7: Remodeling retinal are perfused and sensitive to flow. A) Between post-natal days 3 and 9 (P3-P9), a primitive vascular network (visualized by endothelial-specific markers PECAM1 or isolectin B4 (IB4)) sprouts from a centrally located plexus and expands radially towards the retinal edge. (Colours: PECAM1 (red); Scale bar: 1mm) **B)** At P6, the developing vasculature includes an outer region of active endothelial sprouting and vascular remodeling (R), and an interior “mature” region (M) where smooth muscle actin (SMA)-invested arteries were detected. The R-M boundary (dotted yellow line) was assigned as the point at which SMA investment of arteries is no longer detected above background levels. (Colours: PECAM1 (red), SMA (cyan); Scale bar: 250μm (lower magnification), 50μm (higher magnification); Symbols: a = Artery, v = Vein, R = Remodeling, M = Mature). **C)** Intracardiac injection of rhodamine dextran marked vessels at the R-M boundary (Colours: PECAM1 (red), SMA (cyan), Dextran (green); Scale bar: 250μm; Symbols: a = Artery, v = Vein, R = Remodeling, M = Mature). **D)** Antibodies against phosphorylated Erk5 (pERK5), which occurs in response to laminar shear in cultured endothelial cells, co-labeled endothelial (PECAM1+) cells of the

remodeling plexus. (Colours: PECAM1 (red), SMA (cyan), pERK5 (green); Scale bar: 50μm; Symbols: a = Artery).

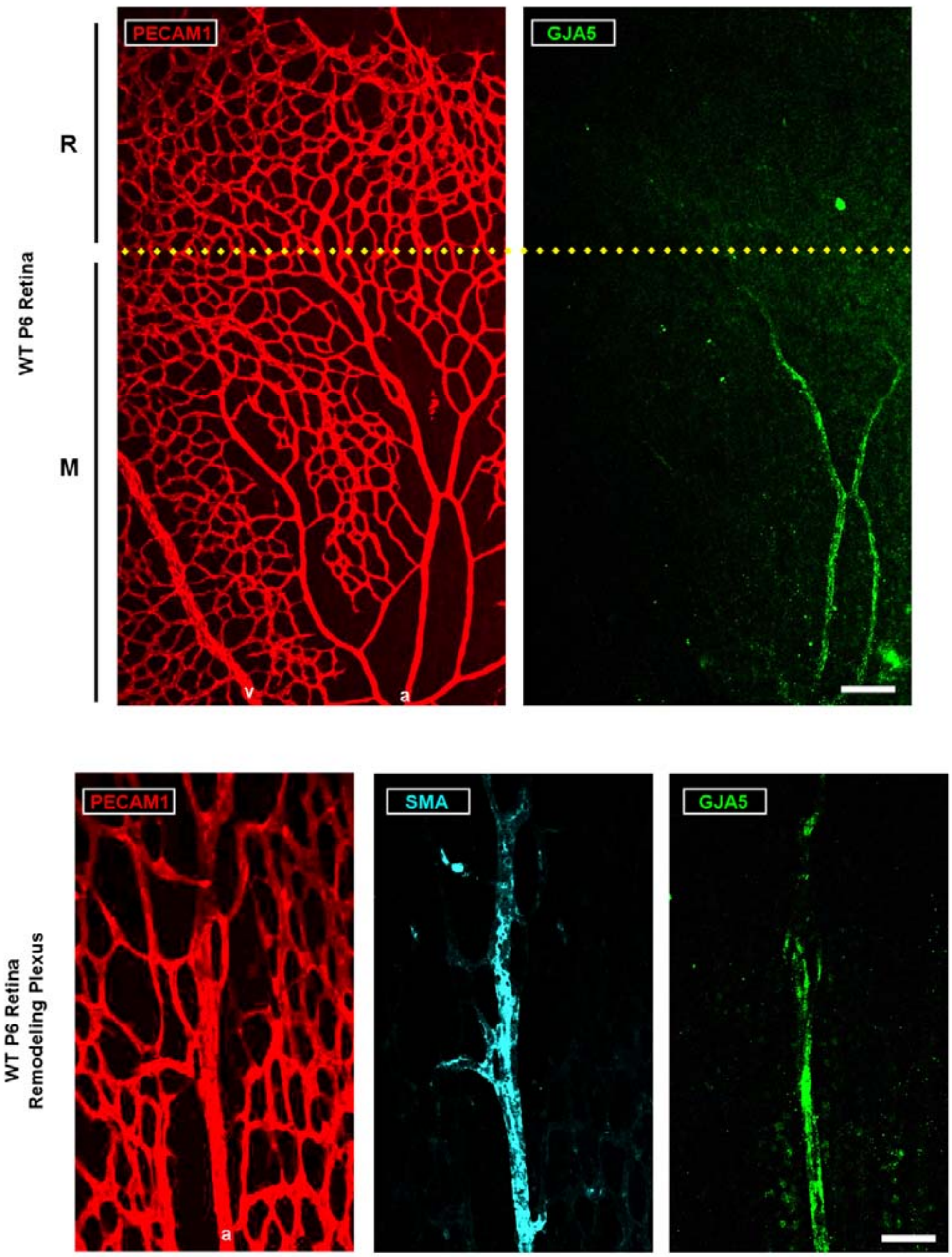

Supplementary Figure 8: GJA5 (Cx40) is limited to SMA-invested arteries. GJA5, a common marker of arterial identity, was limited to expression in arteries of the "mature" (M) vasculature where SMA investment was complete. GJA5 was not detected in remodeling vessels beyond the R-M boundary. (Colours: PECAM1 (red), GJA5 (green), SMA (cyan); Scale bars: 100μm (lower magnification), 25μm (higher magnification);

Symbols: a=Artery, v=Vein, R=Remodeling, M=Mature).

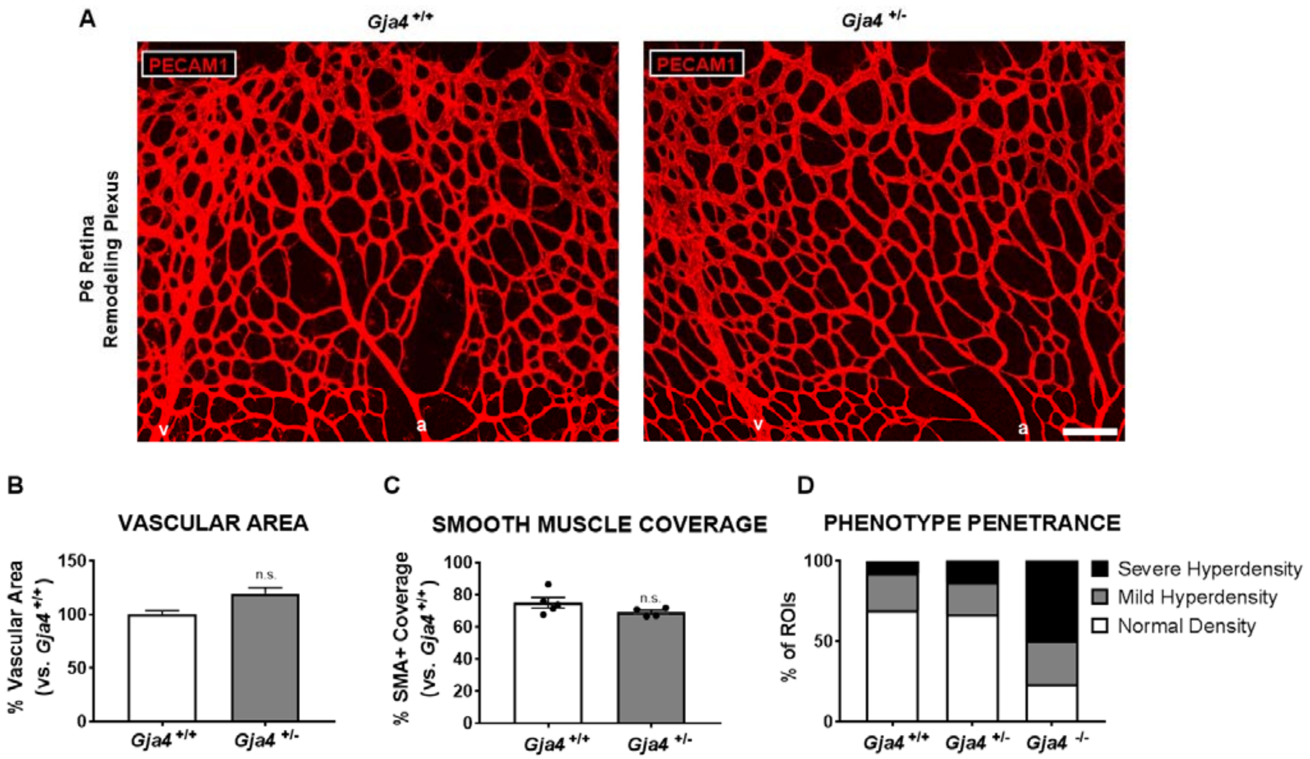

Supplementary Figure 9: P6 retinas of *Gja4*$^{+/-}$ mice are phenotypically indistinguishable from *Gja4*$^{+/+}$ (wild-type) controls, whereas remodeling plexus hyperdensity phenotype of *Gja4*$^{-/-}$ mice has high penetrance. A) No morphological abnormalities of the retinal vasculature were observed between *Gja4*$^{+/-}$ and *Gja4*$^{+/+}$ retinas. (Colours: PECAM1 (red); Scale bar: 100μm; Symbols: a=Artery, v=Vein). Quantification of representative images revealed no differences in either **B)** vascular area (mean % vascular area per retina ± SEM vs. *Gja4*$^{+/+}$; n=5 (*Gja4*$^{+/+}$), n=8 (*Gja4*$^{+/-}$)). **C)** SM investment (mean % SMA+ outgrowth per retina ± SEM vs. *Gja4*$^{+/+}$; n=4 (*Gja4*$^{+/-}$), n=5 (*Gja4*$^{+/+}$)) between P6 *Gja4*+/- and *Gja4*$^{+/+}$ mice. **D)** Mild hyperdensity (>110% of mean density of *Gja4*$^{+/+}$) was observed in 23% of regions of interest (ROIs) from *Gja4*$^{-/-}$, whereas severe hyperdensity (>125% of mean density of *Gja4*$^{+/+}$ animals) was observed in 57% of regions of interest (ROIs) taken from the remodeling plexus of *Gja4*$^{-/-}$ (compared to 8% in *Gja4*$^{+/+}$ and 13% in *Gja4*$^{+/-}$) indicating high penetrance of this phenotype in *Gja4*$^{-/-}$ animals (n=12-35 ROIs from n=4-8 retinas).

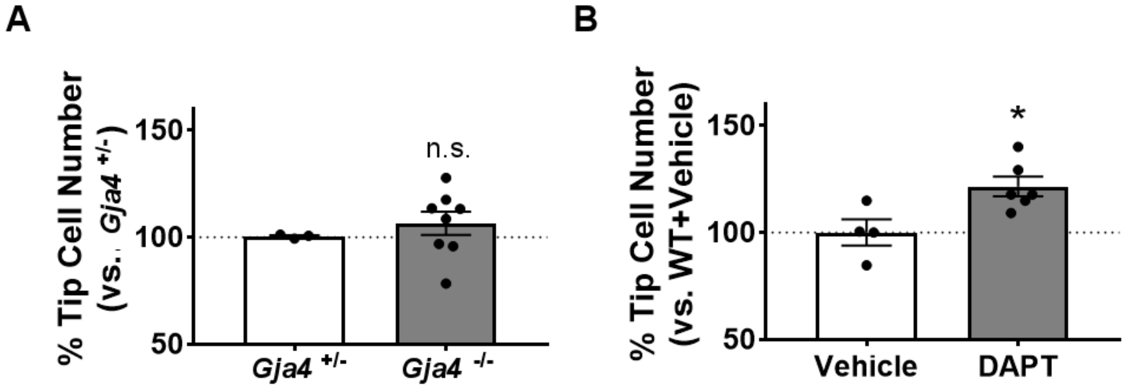

Supplementary Figure 10: Tip cell number is not increased in *Gja4*$^{-/-}$ mice. A) The percentage of tip cell number per retinal ROI is not significantly different between *Gja4*$^{-/-}$ mice and *Gja4*$^{+/-}$ littermate controls (mean tip cell protrusion % per retina ± SEM; n=3 (*Gja4*$^{-/-}$), n=8 (*Gja4*$^{+/-}$)). By contrast, **B)** the percentage of tip cell number per retinal ROI is significantly increased in WT animals upon treatment with 24h DAPT compared to vehicle-treated controls. (mean tip cell protrusion % per retina ± SEM; n=4 (Vehicle), n=6 retinas (DAPT); Students' t-test: p=0.02).

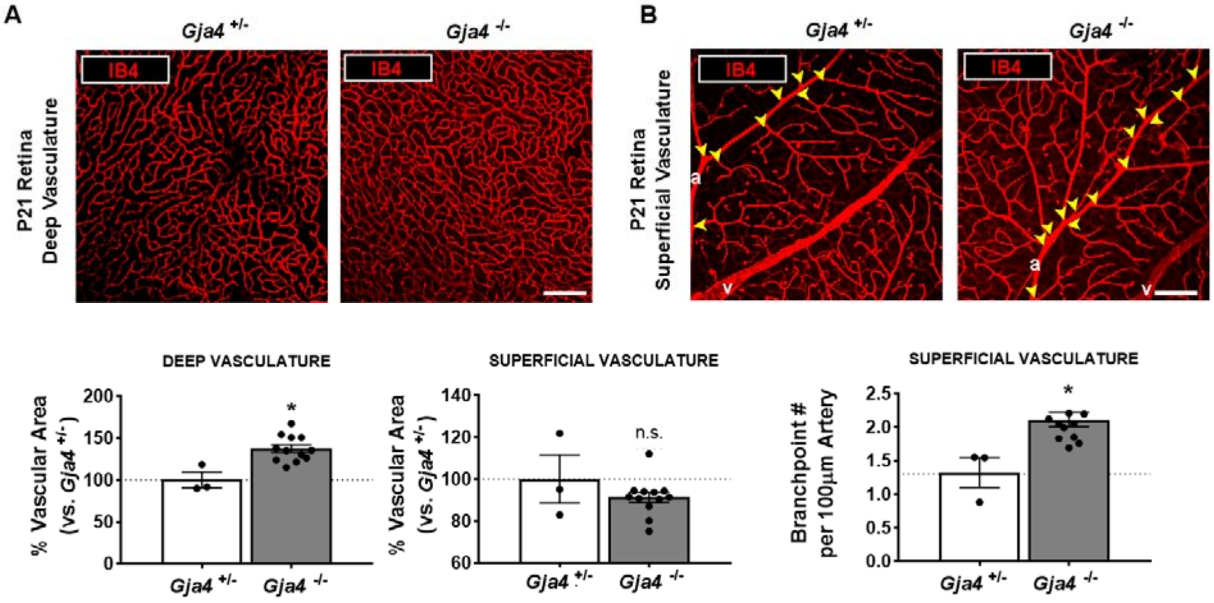

Supplementary Figure 11: Excessive vessels are retained at P21 in *Gja4*$^{-/-}$ animals. A) The deep vascular plexus of P21 *Gja4*$^{-/-}$ animals was significantly more dense compared to littermate *Gja4*$^{+/-}$ controls, whereas no measurable differences in superficial vascular plexus density were observed (Colours: IB4 (red); Scale bar: 50µm; mean % vascular area per retina ± SEM vs. *Gja4*$^{+/-}$; n=3 (*Gja4*$^{+/-}$), n=12 (*Gja4*$^{-/-}$); Students' t-test: p=0.003 (% vascular area, deep)). Nonetheless, **B)** in the superficial vascular plexus, the number of arterial branches (yellow arrowheads) was significantly increased in Cx37$^{-/-}$ mice (Colours: IB4 (red); Scale bar: 50µm; Symbols: a=Artery, v=Vein; mean branchpoint number per 100µm of artery per retina ± SEM vs. *Gja4*$^{+/-}$; n=3 (*Gja4*$^{+/-}$), n=12 (*Gja4*$^{-/-}$); Students' t-test: p=0.006).

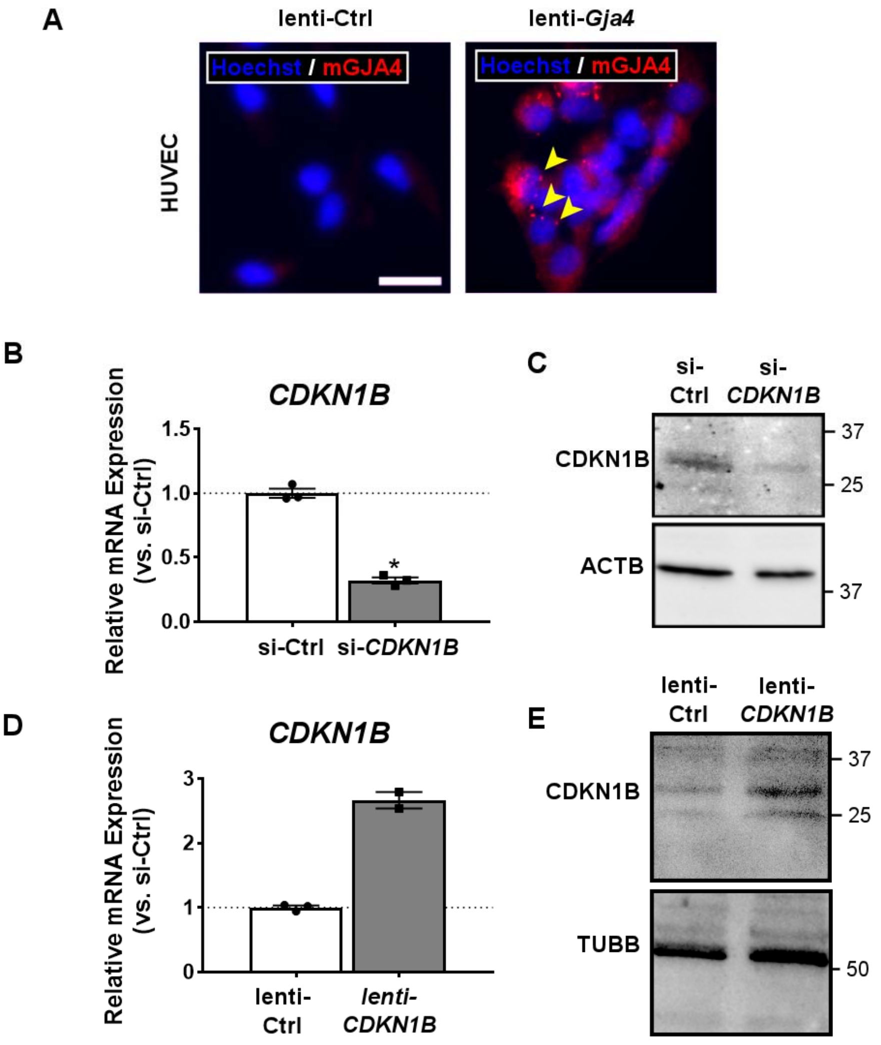

Supplementary Figure 12: Validation of tools used to manipulate GJ4 and CDKN1B expression. A) Following 96h of lentiviral transduction of HUVEC with constitutively-expressed sequence for full-length *mGja4* (i.e. mouse Cx37), numerous mouse GJA4-positive punctae (yellow arrowheads) were detected at intercellular junctions of HUVEC indicating constitutive expression and proper localization, consistent with previous reports for this construct. (Colours: mouse GJA4 (red), Hoechst (blue). Scale bar: 50µm). **B)** Following 120h of transfection with si-*CDKN1B*, endogenous CDKN1B mRNA expression was knocked down by ~70% compared to cells treated with scrambled (si-Ctrl) control sequence (mean relative mRNA expression ± SEM v.s. si-Ctrl; n=3; Students' t-test: $p<0.001$). **C)** CKN1B protein expression was also reduced in si-CDKN1B treated cells vs. control cells. **D)** Following 96h lentiviral transduction with constitutively-expressed full-length human CDKN1B (lenti-*CDKN1B*), human *CDKN1B* mRNA was increased by

nearly three-fold in HUVEC compared to cells treated with empty lentivirus (lenti-Ctrl) (mean relative mRNA expression ± SEM vs. lenti-Ctrl; n=2 (lenti-p27), n=3 (lenti-Ctrl)). **E)** CDKN1B protein expression was also elevated compared to controls.

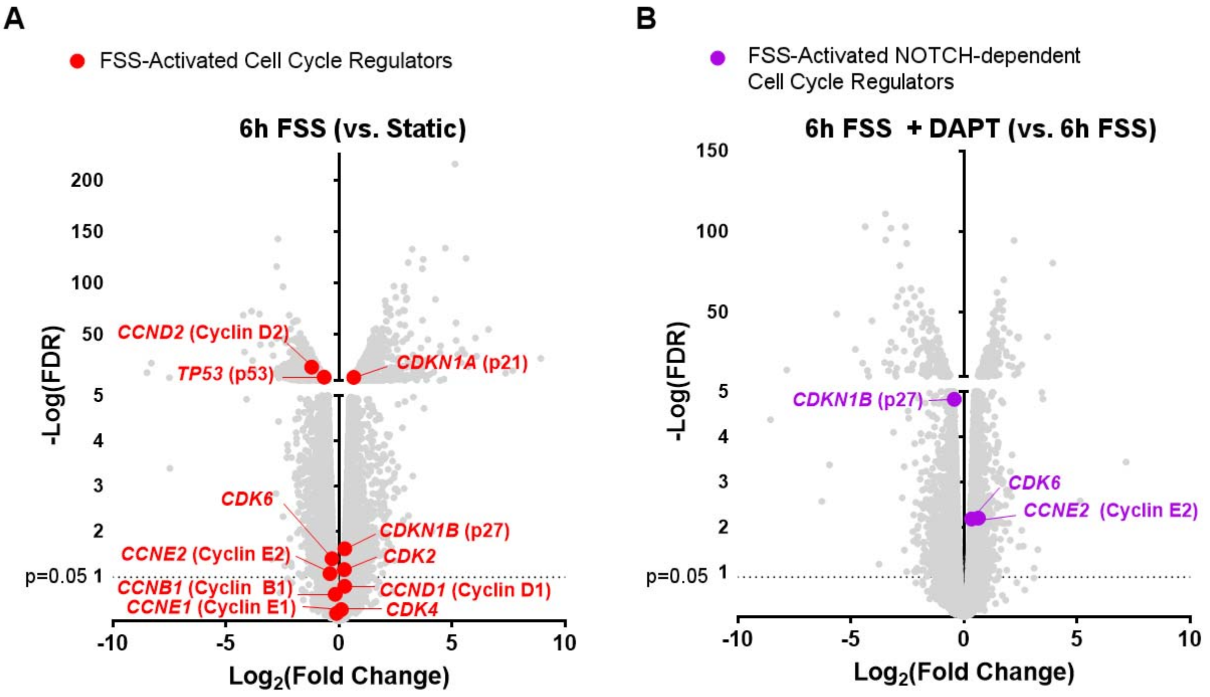

Supplementary Figure 13: NOTCH inhibition under FSS alters cell cycle regulator expression. A) In whole transcriptome analysis of HUVEC exposed to 6h FSS, the mRNA expression of numerous cell cycle regulators were significantly altered (vs. Static). **B)** Of cell cycle regulators whose expression was significantly altered by FSS, only the expression of G1/S transition regulator *CDKN1B* (p27) – as well as its binding partners *CDK6* and *CCNE2* (Cyclin E2) -- was also significantly affected by addition of 10μM DAPT (vs. DMSO) under FSS.

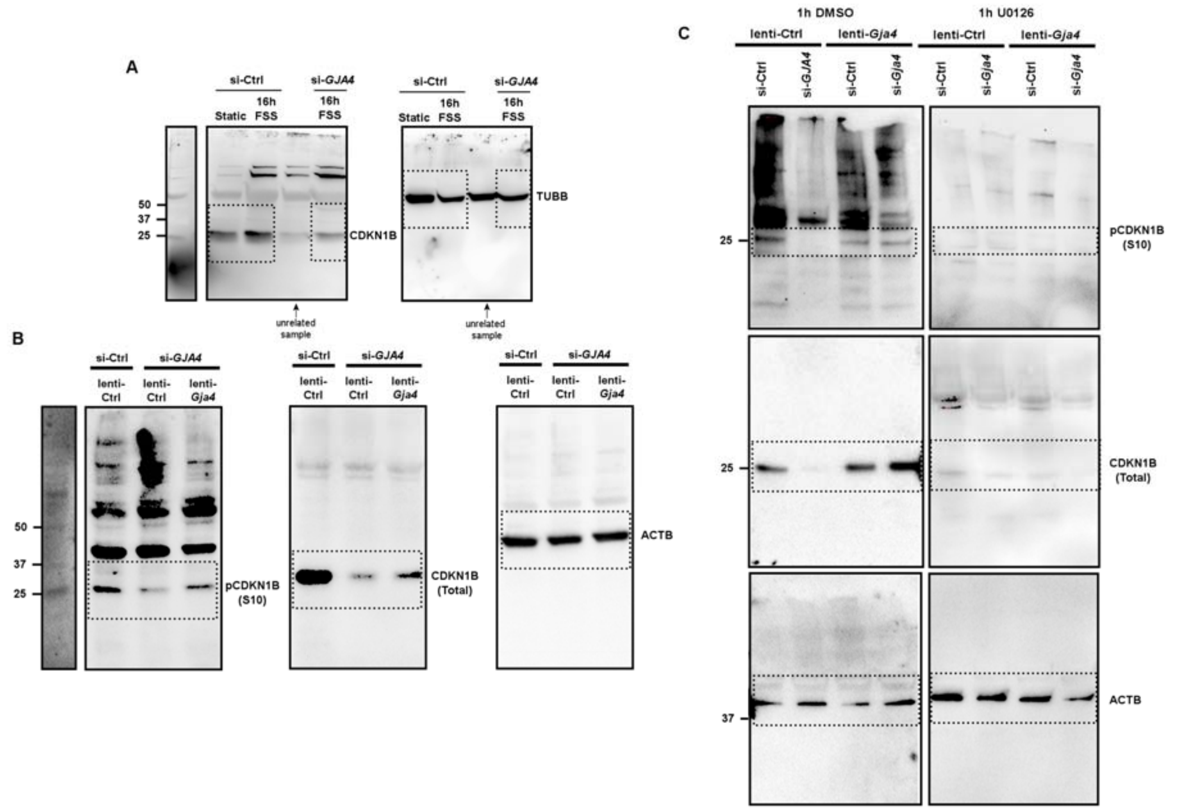

Supplementary Figure 14: Uncropped western blots for Figure 5. A) Uncropped western blots showing that FSS upregulates CDKN1B but that si-*GJA4* blocks this effect. **B)** Uncropped blots showing that si-*GJA4* reduces total CDKN1B as well as S10 phosphorylation of CDKN1B (pCDKN1B-S10), and that rescue of GJA4 expression with lenti-*Gja4* restores CDKN1B expression and phosphorylation. **C)** Uncropped western blots showing that treatment with 1h U0126, an inhibitor of MAPK/ERK signaling, prevents rescue of CDKN1B phosphorylation and total CDKN1B expression by lenti-*Gja4*. Dashed lines indicate how blots were cropped for display in **Figure 5**.

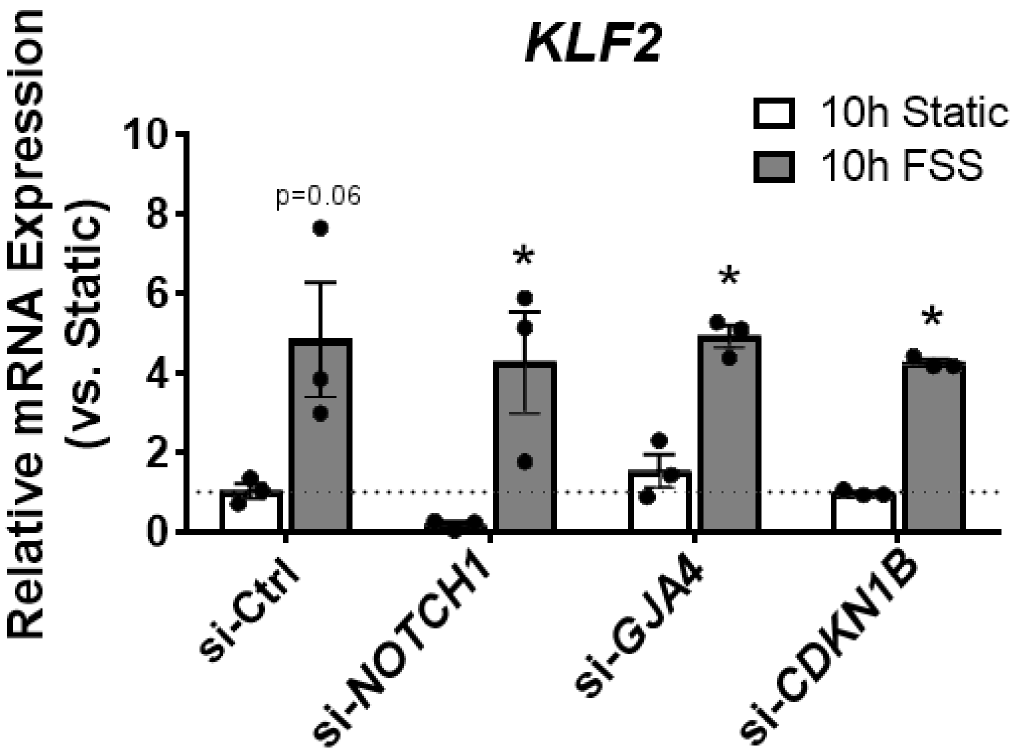

Supplementary Figure 15: Mechanotransduction signaling complexes are not disrupted by knockdown of NOTCH1, GJA4, or CDKN1B. 10h FSS activated *KLF2* expression to comparable levels in si-Ctrl, si-*NOTCH1*, si-*GJA4*, and si-*CDKN1B* groups (mean relative mRNA expression ± SEM vs. si-Ctrol + Static; representative of n=2 experiments; Students' t-test: p=0.03 (si-*NOTCH1*), p=0.002 (si-*GJA4*), p<0.0001 (si-*CDKN1B*)).

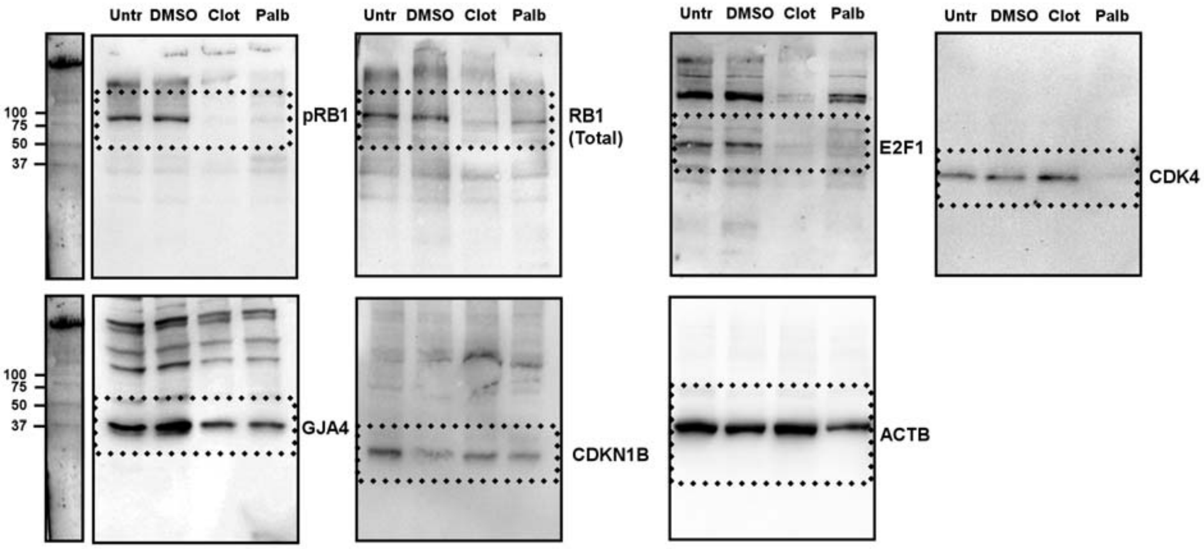

Supplementary Figure 16: Uncropped western blots for Figure 7. Uncropped western blots showing that clotrimazole (Clot) and palbociclib (Palb) reduces phosphorylation of RB1 (pRB1), total RB1, and E2F1 indicating induction of G1 arrest. In addition, palbociclib eliminates CDK4 expression. Neither drug affects GJA4 expression, and has minimal effects on CDKN1B, although with clotrimazole treatment CDKN1B appears slightly elevated. Dashed lines indicate how blots were cropped for display in **Figure 5**.

Supplementary Table 1. Flow-Sensitive Signaling Modules Associated with Subgroup of GO-NGO Terms That Describe Cell Proliferation in the Context of Cell Signaling and Vessel or Tissue Development

Signaling Module Name	Genes in Module Altered by Flow (% of Total Genes in Module)	Average Hits / Gene in GO-NGO Subgroup Gene List
Notch signaling (M00682)	30%	13
TGF-β signaling (M00680)	29%	13
Hedgehog signaling (M00678)	29%	10
MAPK (ERK1/2) signaling (M00687)	29%	9
Activin signaling (M00681)	33%	8
BMP signaling (M00679)	16%	3

Supplementary Table 2. Top Five Genes Altered by NOTCH Inhibition (via DAPT) under 6h Shear

Gene Name (Protein Name)	Log2Fold (vs. Untreated)	FDR
GJA5 (Cx40)	-3.5	6.1×10^{-112}
TMEM100 (Transmembrane Protein 100)	-4.4	1.4×10^{-103}
GJA4 (Cx37)	-2.6	1.9×10^{-103}
SLC46A3 (Solute Carrier Family 46, Membrane 3	-3.2	7.2×10^{-103}
GUCY1A3 (Guanylate Cyclase 1)	-3.5	1.5×10^{-95}

Supplementary Table 3: RBP-Jκ binding sites are detected within the human and mouse *GJA4* gene regulatory regions. *In silico* consensus sequence scanning of -5kb to +3kb of the mouse and human *Gja4* transcriptional start site by DMINDA software revealed numerous high-probability binding sites for RBP-Jκ (with scores corresponding to $p<0.001$).

Start (from TSS)	End (from TSS)	Sequence	Score
		MOUSE	
-4811	-4804	CC**TGGGA**G	10.82
-3101	-3094	CC**TGAGA**A	11.04
-2105	-2098	CC**TGGGA**A	10.93
-153	-146	TC**TGGGA**T	11.00
-71	-64	CC**TGGGA**G	10.82
+445	+495	TC**TGGGA**A	11.12
		HUMAN	
-3175	-3168	CC**TGGGA**G	10.51
-2429	-2421	TC**TGGGA**G	10.91
-971	-964	CC**TGGGA**A	10.69
-299	-292	CC**TGAGA**G	11.00
-284	-277	CC**TGGGA**G	10.51
+2252	+2259	CC**TGAGA**A	10.87
+2269	+2276	CC**TGGGA**T	10.76

Supplementary Table 4. Oligonucleotide sequences used in this study. All sequences are listed in 5’ to 3’ orientation.

Gene Name (Protein Name)	Forward Sequence	Reverse Sequence
ACTB (human β-Actin)	TCACCCACACTGTGCCCATCTACGA	CAGCGGAACCGCTCATTGCCAATGG
Actb (mouse β-Actin)	AGAGGGAAATCGTGCGTGAC	CAATAGTGATGACCTGGCCGT
Ccnd1 (mouse CCND1)	GCGTACCCTGACACCAATCTC	CTCCTCTTCGCACTTCTGCTC
Ccne1 (mouse CCNE1)	GTGGCTCCGACCTTTCAGTC	CACAGTCTTGTCAATCTTGGCA
Ccne2 (mouse CCNE2)	ATGTCAAGACGCAGCCGTTTA	GCTGATTCCTCCAGACAGTACA
CDK4 (human CDK4)	ATGGCTACCTCTCGATATGAGC	CATTGGGGACTCTCACACTCT
Cdk4 (mouse CDK4)	GGACAGGTAGCGATCCAGGT	TCCTCCATTAGGAACTCTCACAC
Cdk6 (mouse CDK6)	GGCGTACCCACAGAAACCATA	AGGTAAGGGCCATCTGAAAACT
Cdkn1a (mouse CDKN1A) (p21)	CCTGGTGATGTCCGACCTG	CCATGAGCGCATCGCAATC
CDKN1B (human CDKN1B) (p27)	ATCACAAACCCCTAGAGGGGCA	GGGTCTGTAGTAGAACTCGGG
Cdkn1b (mouse CDKN1B) (p27)	TCAAACGTGAGAGTGTCTAACG	CCGGGCCGAAGAGATTTCTG
Cdkn2a (mouse CDKN2A) (p16)	AACTCTTTCGGTCGTACCCC	GCGTGCTTGAGCTGAAGCTA
EPHB2 (human EPHB2)	TATGCAGAACTGCGATTTCCAA	TGGGTATAGTACCAGTCCTTGTC
GAPDH (human GAPDH)	ACAACTTTGGTATCGTGGAAGG	GCCATCACGCCACAGTTTC

Gapdh (mouse GAPDH)	AGGTCGGTGTGAACGGATTTG	TGTAGACCATGTAGTTGAGGTCA
GJA4 (human GJA4) (Cx37)	ACACCCACCCTGGTCTACC	CACTGGCGACATAGGTGCC
GJA4 promoter (human GJA4) (Cx37)	GAGAGAGAACAGAGGAGAGGAA	GAGGTGCAGACTCCCTAAAC
Gja4 (mouse GJA4) (Cx37)	CCCACATCCGATACTGGGTG	CGAAGACGACCGTCCTCTG
GJA5 (human GJA5) (Cx40)	CCGTGGTAGGCAAGGTCTG	ATCACACCGGAAATCAGCCTG
Gja5 (mouse GJA5) (Cx40)	CCACAGTCATCGGCAAGGTC	CTGAATGGTATCGCACCGGAA
HES1 (human HES1)	TCAACACGACACCGGATAAAC	GCCGCGAGCTATCTTTCTTCA
Hes1 (mouse HES1)	CCAGCCAGTGTCAACACGA	AATGCCGGGAGCTATCTTTCT
HEY1 (human HEY1)	GTTCGGCTCTAGGTTCCATGT	CGTCGGCGCTTCTCAATTATTC
Hey1 (mouse HEY1)	GCGCGGACGAGAATGGAAA	TCAGGTGATCCACAGTCATCTG
HEY2 (human HEY2)	GCCCGCCCTTGTCAGTATC	CCAGGGTCGGTAAGGTTTATTG
Hey2 (mouse HEY2)	AAGCGCCCTTGTGAGGAAAC	GGTAGTTGTCGGTGAATTGGAC
NOTCH1 (human NOTCH1)	GAGGCGTGGCAGACTATGC	CTTGTACTCCGTCAGCGTGA
NOTCH4 (human NOTCH4)	TGTGAACGTGATGTCAACGAG	ACAGTCTGGGCCTATGAAACC
Trp53 (mouse TRP53) (p53)	GTCACAGCACATGACGGAGG	TCTTCCAGATGCTCGGGATAC
