# Supplementary material for: Shear-induced Notch-Cx37-p27 axis arrests endothelial cell cycle to enable arterial specification
Source: Nat Commun. 2017 Dec 15;8:2149. doi: 10.1038/s41467-017-01742-7 (PMC5732288; doi:10.1038/s41467-017-01742-7)
Supplement: Supplementary file 2 — Description of Additional Supplementary Files [file 41467_2017_1742_MOESM2_ESM.pdf]

Description of Additional Supplementary Files

File Name: Supplementary Data 1
Description: **Subgroup of GO-NGO Pairs that Describe Cell Proliferation in the Context of Cell Signaling and Vessel Development**. Selected list of statistically enriched nested GOSeq (NGOSeq) terms associated with upper-level GOseq terms detected in whole transcriptome analysis of HUVEC plated under 6h static vs. 6h shear conditions and that were related to cell proliferation, cell signaling, and vessel development.
